# A single dose of cannabidiol (CBD) positively influences measures of stress in dogs during separation and car travel

**DOI:** 10.3389/fvets.2023.1112604

**Published:** 2023-02-22

**Authors:** Alysia B. G. Hunt, Hannah E. Flint, Darren W. Logan, Tammie King

**Affiliations:** Waltham Petcare Science Institute, Waltham on the Wolds, United Kingdom

**Keywords:** cannabidiol, CBD, dog, stress, anxiety, behavior, animal welfare

## Abstract

Many dogs experience stress when separated from their caregivers, as well as when traveling in vehicles. Pet owners employ various approaches to managing these issues, from training, to giving medications and supplements, often with mixed results. Cannabidiol (CBD) can alleviate stress and anxiety in humans but the effect it has on canine stress is less well-documented. The present study aimed to understand the impact of being left alone and traveling in a car on measures of canine stress, and establish whether a single dose of a tetrahydrocannabinol (THC)-free CBD distillate could positively influence any measures of stress. In a blinded, parallel design study, a population of dogs were either left alone in a familiar room (*n* = 21) or underwent a short car journey (*n* = 19). A range of physiological and behavioral measures were collected pre, during and post-test. Significant changes in several stress-related measures (serum cortisol, mean ear temperature, heart rate, heart rate variability, whining and a stressed/anxious behavioral factor) were observed from baseline to test, with the car journey test paradigm eliciting a more pronounced stress response overall. The mitigating effect of CBD treatment varied by measure and test, with some indicating a significant reduction in canine stress compared to the placebo group. Additional research is required to fully understand the complex effect of CBD on canine wellbeing.

## Introduction

Pet dogs are likely to experience a variety of stress-inducing scenarios over their lifetime, often due the relationship they have with people (1, 2). These are often unavoidable and are commonly related to events such as car travel (3, 4) and separation from caregivers or conspecifics (5–8). Due to the ever-changing human-pet relationship, there is an increasing requirement for dogs to fit into human lifestyles which can cause stress and anxiety to both people and their pets. Although mild stress is not considered harmful (9), welfare is compromised if an animal experiences high levels of acute stress or is exposed to prolonged stressors (10) which can lead to chronic physical and emotional health issues (11). As a social species, dogs form close bonds with humans and other animals. When socially isolated or separated from bonded individuals for prolonged periods of time, separation-related behaviors may be exhibited (8, 12, 13). This can manifest into separation-related anxiety which is one of the most reported stress-related issues in pet dogs, making up to 50% of referral cases to behaviorists (5, 8, 13–15). Additionally, anthropomorphism following an owner's absence can lead to humans misinterpreting dog emotional states, which can further negatively impact pet welfare (16). Dog owners are often left feeling frustrated due to the damage pets cause to their property, as well as not being able to successfully manage the problem behavior. This can negatively impact the human-animal bond and often leads to dog relinquishment (7). Following the recent COVID-19 pandemic, the incidence of separation-related behaviors in dogs has increased and cases are expected to continue to rise (12, 17, 18).

Another common situation that can elicit stress in dogs and results in negative emotional states, is car travel (19–21). Most dogs will experience traveling in a vehicle during their lifetime, the frequency of which is dependent upon the owners' lifestyle, demographic, and geographical distance to places such as parklands, veterinary clinics, and dog husbandry professionals (e.g., groomers, dog boarding facilities, dog daycare). Transportation is stressful for some animals due to the intense combination of auditory and visual stimuli experienced in a moving vehicle (22). It is reported that one in four dogs suffer from travel-related problems (4) and commonly display behaviors including trembling, panting, shaking, hypersalivating, self-licking, and barking/whining (3, 23). Notwithstanding the dog's emotional and physical discomfort, these behaviors could distract the driver, possibly endangering the pet, caregiver, and other road users. Despite the relative frequency of people transporting dogs and the welfare implications this may have on pets and humans, the transport of companion animals has not been widely researched. As such, a better understanding of the effects car travel has on pet dogs, along with ways in which people can support dogs during journeys in the car would be of value.

Current research into treatments aimed at alleviating stress in dogs during periods of separation and during transportation are generally focused on the efficacy of pharmacological interventions (24, 25), alone or in combination with behavioral modification programs (5). Clomipramine is a commonly used anti-depressant to treat separation-related disorders and has been shown to reduce destructive behavior, as well as inappropriate defecation and urination, but not the prevalence of vocalizations (25). Reported side effects include vomiting and sedation (25). Similarly, fluoxetine administration can reduce separation-related behaviors but may also cause lethargy, inappetence, seizures and depression in some dogs (24, 26). Many of these drug interventions require daily administration for several weeks before a notable effect is observed (27) and reported efficacy is inconsistent (28). Pheromone based substances are also commercially available, are considered safe and convenient, and have been shown to reduce some signs of canine stress during car travel and during periods of separation, though these responses are not uniform across all individuals (3, 4, 29).

An intervention which is gaining popularity is the administration of cannabidiol (CBD), likely in response to it being perceived as a natural treatment among pet owners (30). CBD is a non-psychoactive phenolic cannabinoid typically derived from the processing of hemp *(Cannabis sativa L.)*. It has been demonstrated to have positive effects on human and non-human animal health and wellbeing (31, 32) *via* the endocannabinoid system, which is responsible for modulating pain and inflammatory processes (33). CBD has been shown to down-regulate stress-related signals that can lead to chronic inflammation and some pain responses in humans (34). Emerging evidence suggests CBD may also be efficacious in the treatment of anxiety in humans (35, 36). Precisely how CBD mediates these effects is both complex and not fully understood. It acts as an allosteric modulator of the CB1 and CB2 receptors present in nociceptive and pain neurons, but also as a potent agonist and antagonist of other G-protein coupled receptors in the immune and central nervous systems. CBD is also increasingly used within the veterinary industry (37) and it is reported that short term oral dosing of CBD up to 20 mg/kg daily (38) and single oral gavage dosing of 62 mg/kg does not cause any significant adverse effects in healthy dogs (39). Additionally, a recent 6-month safety study found 4 mg/kg oral daily dosing (40) was well-tolerated suggesting CBD is safe for healthy dogs when fed appropriately. However, less is empirically known regarding the efficacy of this compound in dogs, with the majority of clinical studies examining the efficacy of CBD in alleviating the symptoms of pain resulting from osteoarthritis (37, 41, 42). There is limited evidence supporting the efficacy of CBD in alleviating stress or anxiety in pets, with previous studies not demonstrating a positive effect (43). Nevertheless, there are numerous commercially available products on offer to pet owners that claim to support dog emotional health.

The aim of this study was to address two key objectives. Firstly, to understand the impact a separation event and car travel has on canine stress, using a combination of subjective and objective measures. Secondly, to evaluate the effect of a single dose of a THC-free broad-spectrum CBD distillate on measures of canine stress during these two events. We hypothesized that at least one of the events would elicit behavioral and physiological measures of stress in dogs, and that a single administration of CBD would have a positive effect on those measures.

## Materials and methods

### Animals and husbandry

Forty healthy, adult dogs, twenty-two males and eighteen females of three breeds (17 Labrador Retrievers, 8 Beagles, and 15 Norfolk Terriers), with a mean age of 4.1 years (ranging from 1.2 to 9.4 years) participated in the study. All dogs were housed in pairs within kennels at the Waltham Petcare Science Institute (Leicestershire, UK) with free access to indoor and outdoor environments. Dogs were provided with comprehensive training and socialization programs, adjusted to the needs of the individual dogs as per the Institute's pet keeping requirements. Prior to the study, dogs were habituated to the testing environments and associated equipment and underwent appropriate training to facilitate sample collections (e.g., blood samples). Dogs were weighed to establish an accurate dose of CBD relative to individual bodyweight. The targeted oral dose for each dog was 4 mg/kg bodyweight with an acceptable range of 3.38–4.44 mg/kg bodyweight (40). This study was approved by the Waltham Animal Welfare and Ethical Review Body (80265) and conducted under the authority of the Animals (Scientific Procedures) Act 1986.

### Study design

**Figure 1:**
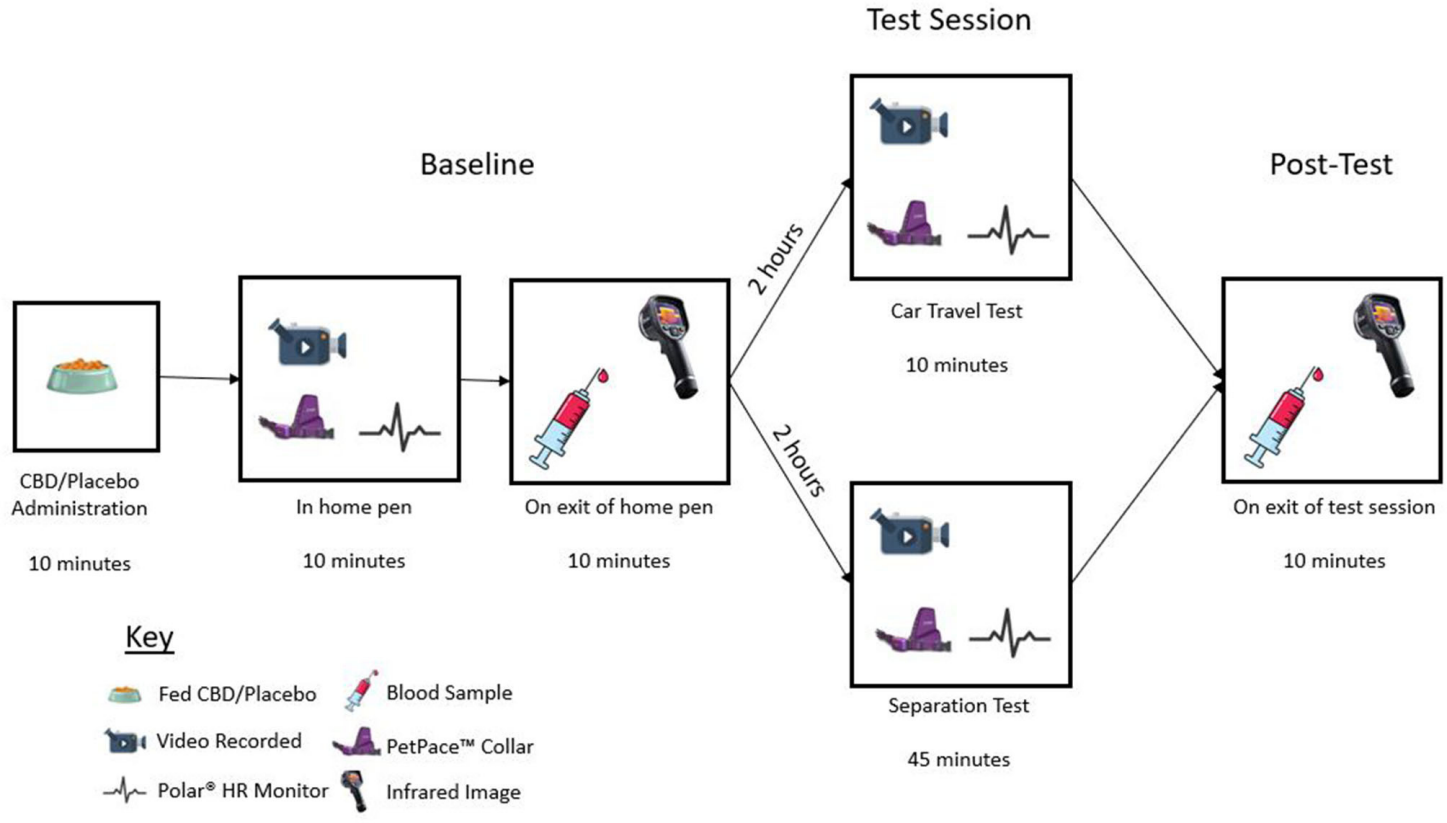
Diagram illustrating test day timeline, including CBD/placebo administration, baseline recording, baseline sample collection, test session, and post-test sample collection.

A blinded, parallel design study was conducted at the Waltham Petcare Science Institute to determine the anxiolytic effects of CBD in parallel with a long-term safety study (40). Over this 6 month study dogs were given daily administration of CBD and indicators of stress were measured following exposure to acute and chronic stress-inducing events. Here we report findings from the first CBD or placebo dosing, immediately followed by a single exposure to the two testing scenarios. Dogs were randomized and balanced across two groups: placebo control (*n* = 20) and treatment (*n* = 20). Dogs were further randomized into two groups to denote the type of testing paradigm they would experience within those groups. The parameters age, sex, breed, and housing location were considered when balancing the groups. Prior to the study, one dog was identified as having mobility concerns and so was unable to safely enter the car. Therefore, this dog was moved into the separation group resulting in 21 dogs (10 control and 11 treatment) experiencing a separation event and 19 dogs (10 control and nine treatment) experiencing car travel. Dogs in the separation group were habituated to the testing room until they were deemed comfortable in the environment with their experienced handler present. Dogs in the car travel group were trained to enter a crate within the car *via* a ramp or box setup voluntarily and habituated to the crate. All testing was conducted by two blinded researchers who were responsible for ensuring the event conditions were standardized. Various physiological and behavioral measures were collected *via* wearables, video cameras, and blood sampling prior to, during and directly after the test sessions (Figure 1). Dogs were closely monitored throughout each test session for signs of distress and/or compromised welfare based on pre-defined removal criteria.

### Collection of baseline measures

Prior to their morning meal, each dog had a Polar^®^ H10 monitor (Polar Electro, Kempele, Finland) placed on their chest with ultrasound gel applied to the sensor. Additionally, a PetPace™ collar (Burlington, MA, USA) was secured on the dog's neck. Dogs were left in their home pen, for 10-min and recorded with video cameras (Logitech 920 Webcam, Logitech, Lausanne, Switzerland) placed outside the kennel door. The dog was then led from the pen to the sample room where a baseline thermal image was captured of the dog's face, and a blood sample (described in detail below) was collected within a 10-min window.

### Treatment

Dogs received either a placebo or CBD capsule (~4 mg/kg bodyweight) within a Royal Canin^®^ Pill Assist pocket, with their morning meal. Hemp-derived distillate and placebo oils were acquired from Canopy Growth Corporation (Ontario, Canada) and processed by Kazmira LLC (Colorado, USA). The distillate was diluted with a food-grade sunflower oil and manufactured in soft gel capsules (bovine origin; RNA Corporation, Illinois, USA). Capsules were then analyzed for potency and purity as previously described (40), finding only trace amounts of non-psychoactive cannabidivarin (0.004 mg CBDV/mg of CBD) in a small number of capsules, and no detectible THC or other cannabinoids. The placebo soft gel capsules were manufactured to match the conformity of the CBD-containing capsules, minus the CBD, to maintain the blinding of the study. Two hours after placebo/CBD administration had been confirmed, dogs were exposed to either the separation event or car travel.

### Separation event

**Figure 2:**
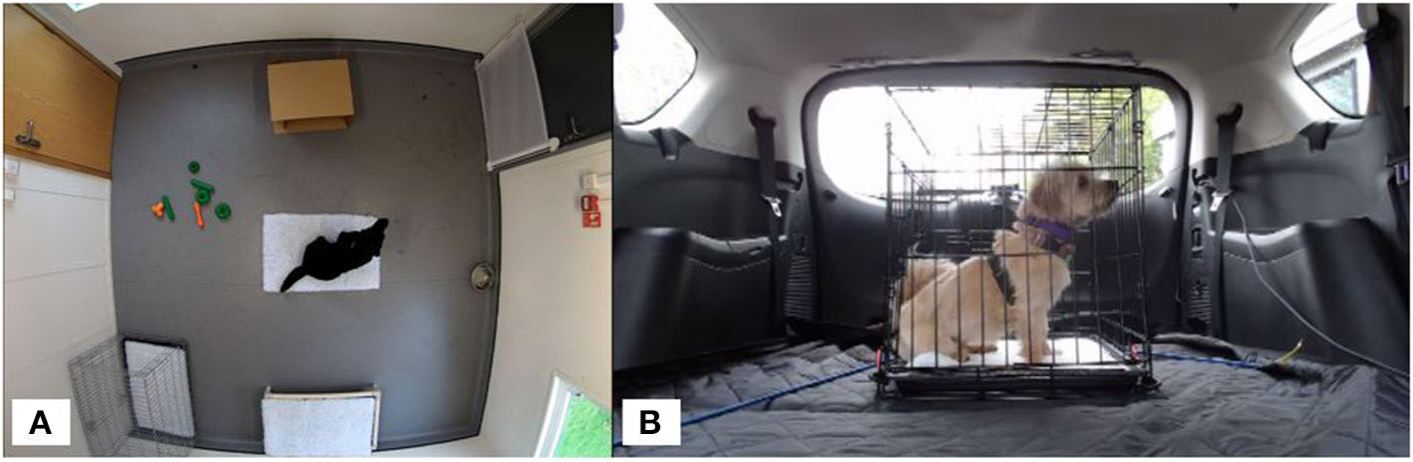
**(A)** Separation test room setup showing position of vet bedding (center, with a dog on top), then clockwise from top: cardboard box, water bowl, dog bed, crate, and toys. **(B)** Car test setup showing metal crate secured in rear of the car. Video cameras were positioned at the front and rear of the crate.

The separation test room (3.71 × 3.58 m) was fitted with a CCTV system (Dauhua 4 k ultraHD IR turret network camera × 4; Reflex Systems, UK). During testing, the room temperature was maintained at 19 ± 2°C. The room contained a metal dog crate with a single piece of vet bedding, a raised hammock bed covered with a piece of vet bedding, a piece of vet bedding in the center of the room, a cardboard box (a common enrichment for this population of dogs) relative to the size of the dog being tested, a filled water bowl and a selection of hard rubber toys (Figure 2A). Once inside, the handler removed the lead or harness from the dog and exited the room. Dogs were left alone for 45-min while being monitored by a researcher in an adjacent room *via* the CCTV system. After 45-min, the handler returned and placed the dog back on lead. The dog was led to the adjacent room for post-test sampling. Dogs were then returned to their familiar housing.

### Car travel

A Ford S-Max mini-van vehicle was used for each car test with a metal crate, appropriate for the dog's size, placed on the top of folded down rear seats inside the vehicle. The crate was fixed in place with bungee cords and contained a single piece of non-slip vet bedding (Figure 2B). Entry to the car was either *via* a non-slip ramp or a step. Once the dog was inside the secured crate, the car boot was closed, and the researcher sat in the driver's seat. Testing took place over Winter (January/February) and the interior of the car was temperature controlled at 19 degrees Celsius. Dogs underwent a standardized 10-min car journey consisting of a range of maneuvers such as a sharp U-turn and a three-point-turn. The speed of the car never exceeded 10 mph due to being in a private enclosed car park area. Dog behavior was recorded using Mediarecorder software (Noldus, Netherlands) *via* two cameras (Logitech 922 webcam; Logitech, Lausanne, Switzerland) mounted in the rear of the car, at either end of the crate, connected to a laptop computer on the front passenger seat. Dogs were monitored periodically *via* the rear-view mirror with the driver remaining silent. On completion of the journey, the dog was removed from the car by their handler and led to a room for post-test sampling. Dogs were then returned to their familiar housing.

### Measures

#### Eye, ear, and nose temperature

At baseline and post-test, a portable infrared camera (FLIR T840, FLIR, Oregon, USA) was used to capture infrared images during the study with a thermal range of −20–150°C and a resolution of 464 × 348 pixels. The value of emissivity was set at 1 as per manufacturer guidelines, replicating previous studies (44, 45). The camera was positioned on a tripod in-line with the head of the dog, lens parallel to the floor and ~1-meter away. Images were analyzed using FLIR Tools software (FLIR; Oregon, USA) as previously described by King et al. (46). Briefly, ellipses were drawn around the anterior surface region of the eye, the exterior surface of the ear flap and the anterior surface of the nose. Mean temperature readings were captured within each ellipse (46, 47).

#### Serum cortisol, immunoglobulin A, and glucose

A small patch of hair was shaved from the dog's neck and topical anesthesia (Ethycalm Plus™; Invicta, West Sussex, UK) was applied to the area. Then a 1.2 ml blood sample was collected from the jugular vein. Blood samples were collected into a clot activating serum tube and kept on ice until analysis and aliquoting was performed within 60-min of sampling. Aliquots for cortisol and IgA were stored at −20°C in preparation for later analyses. The R&D Systems, Parameter™ cortisol immunoassay (bio-techne, Minneapolis, USA) was used following the manufacturer's protocol with an intra-assay variation of <10%. The Abcam, IgA Dog ELISA kit (Boston, USA) was used following the manufacturer's protocol with an intra-assay variation of <10%. A Beckman Coulter (California, USA) kit was used following the manufacturer's protocol to analyze glucose with an intra-assay variation of <3%.

#### Heart rate, heart rate variability, distance traveled, body position, and activity

Heart rate and distance traveled were collected using Polar^®^ H10 heart monitors (Polar Electro, Kempele, Finland) secured around the dog's chest. The heart rate data were also converted into HRV [measured as root mean square of successive differences between normal heartbeats (RMSSD)] by estimating the between-beat time differences from the HR measurements. PetPace™ (Burlington, MA, USA) monitors were used to collect activity (48) and body position data denoting whether the dog was standing, sitting or lying down.

#### Dog behavior

Dog behavioral data from baseline and during the two testing sessions were collected from video footage by trained observers. A series of behavior attributes were scored using a Qualitative Behavior Assessment (QBA), and an ethogram was used to code a separate set of dog behaviors. All QBA scales and ethograms were utilized at both baseline and during test. Baseline videos were 10-min in length; car travel 10-min and for the separation videos the first 5-min, middle 5-min and last 5-min were selected to create a 15-min video to be scored by raters using both the QBA scales and coded ethograms. A QBA previously developed to evaluate welfare of shelter dogs and dogs in a mock veterinary setting (46, 49) was modified for the purpose of this study to include terms more relevant to the specific scenarios. The same 16 terms were used for both the car travel and separation test, with the addition of the term “nauseous” to the car travel QBA (Table 1). Two trained raters who were familiar with dog behavior and blinded to the treatment, scored all videos at each time point. Each rater completed an online form for each assigned video, which comprised of a Visual Analog Scale (VAS) ranging from 0 to 125 placed next to each term. The left end of the VAS scale corresponded to the minimum score (0), meaning the expressive quality indicated by the term was entirely absent in the dog, whereas the right end of the scale represented a maximum score (125), meaning that the quality indicated by the term was strongly present in that dog. Raters were instructed to watch the videos and select a point along the VAS which they felt was appropriate for each term immediately after the video had finished. To assess intra-rater reliability of the QBA terms, each rater re-scored a random selection of 10 videos.

**Table 1:** Terms included in the Qualitative Behavioral Assessments (QBA) used to measure dog behavior during baseline, the separation, and car travel tests.

**Term**	**Definition**
Anxious	Worried, unable to settle or cope with the environment, apprehensive
Alert	Vigilant, inquisitive, on guard
Calm	Absent of strong positive/negative emotions
Comfortable	Without worries, settled in environment, peaceful with external stimuli
Depressed	Dull, sad demeanor, disengaged from and unresponsive to the environment, quiet, apathetic
Explorative	Confident in exploring the environment or new stimuli, investigative
Fearful	Timid, scared, shows postures typical of fear
Lethargic	Sluggish, inactive, unresponsive or slow to respond to external stimuli
Nauseous (car test only)	Salivating, lip licking, facial tension, excessive swallowing, retching, hunched body posture
Nervous	Uneasy, agitated, shows fast arousal, unsettled, restless, hyperactive
Reactive	Responsive to external stimuli
Relaxed	Easy going, calm with no visual evidence of tension in the body
Restless	Unable to rest or relax
Sad	Unhappy, downcast
Stressed	Tense, shows signs of distress
Tense	Stiff, rigid posture, on edge
Uncomfortable	Uneasy, nervous, tense, restless

#### Coded behaviors

Two ethograms were created which included a series of behaviors to be scored, incorporating the evaluation of both events (frequency measures) and states (duration measures). Behaviors were scored from video footage of the dogs during the separation event and during car travel (Table 2). To code the footage collected at baseline, the ethogram for that dogs' specific test session was used, e.g., for a dog who experienced car travel, the car travel ethogram was used to code the baseline footage also. Three trained raters coded each behavior using the relevant ethograms. All raters were assessed for inter and intra-rater reliability at the start of the study on a subset of videos coded three times by each rater. Intra-class correlation coefficients (ICC) were calculated using mean rating (k = 3), two-way mixed effects models, with absolute agreement used for intra-rater reliability and consistency agreement used for inter-rater reliability (50). Good to excellent inter- and intra-rater reliability (ICC > 0.75) (50) was achieved for all coders and behaviors and therefore they were deemed sufficiently reliable for further coding and analysis. A total of 320 videos were randomly assigned between the three raters. All videos were scored using “*The Observer XT 15”* software (Noldus, Netherlands, Europe).

**Table 2:** Ethogram used to measure dog behavior during the separation and car travel tests.

**Behavior**	**Type**		**Definition**
Repetitive pacing/circling	State	Start	Repeats behavioral sequence 2 or more times without a specific goal, following a fixed route. May pause for up to 2 s
		Finish	Dog ceases the repetitive nature of the movement or begins a different behavior
Panting	State	Start	Increased shallow respiration through an open mouth, may have tongue out (70)
		Finish	Mouth is closed—normal breathing resumes
Whining	State	Start	Dog produces sound such as whines, whimpers, and yelps originating from the throat and mouth
		Finish	Sounds production ceases
Barking	Event		Head and lips forward, mouth opening and shutting repeatedly to emit a large, sharp, short sound from the throat (84)^*^
Howling	Event		Raised muzzle perpendicular to ground and emits a long drawn out sound through semi-closed jaw (84)^*^
Play behavior	State	Start	Interaction (e.g., mouthing/pawing) with toys and/or box whilst exhibiting soft/relaxed body language
		Finish	Dog ceases behavior
Digging	State	Start	Mouth/front paws and claws used to attempt movement/displacement of substrate other than external door
		Finish	Dog ceases behavior
Escape behavior	State	Start	Tries to dig, bite, or scratch at the external door—not directed at themselves
		Finish	Dog ceases behavior
Elimination	Event		Squat/leg raised in order to urinate and/or hind end lowered and back arched in order to defecate (84)^*^
Vomiting	Event		Open mouth and retch causing vomit from the mouth
Yawning (car test only)	Event		An involuntary take of breath through a wide-open mouth (70)
Lip licking (car test only)	Event		Dog flicks tongue around the outside of mouth, on lips and/or quickly over the nose

* Modified.

### Statistical analysis

To assess the impact of the stress tests and CBD administration on measures of stress linear mixed effect models were used. The value of the measures was the response, treatment, timepoint and their interaction were the fixed effects, and individual dog ID was the random effect. Three models were run for each measure, with analyses looking separately at car and separation stress tests, as well as at both stress tests combined. The model assumptions were assessed *via* visual inspection of the residuals, and variables were log transformed if they violated model assumptions. The estimated means (back-transformed for log-transformed models) and 95% confidence intervals (95% CI) were extracted from the model. Contrasts were performed between treatments at each timepoint, between timepoints for each treatment, and for the change between timepoints for each treatment with the p-values adjusted using the “Tukey” method to account for multiple pairwise comparisons.

A principal components analysis (PCA) of all QBA terms was conducted excluding terms that had more than 75% of occurrences scored as zero. The weightings from this analysis were used to generate component scores for the relevant identified components. As each video was scored by two raters, a mean score for each of the individual QBA terms, and the relevant component scores were generated for the purposes of analysis. These mean scores were then modeled as above using linear mixed effects models. Inter and intra-rater reliability of the individual QBA terms and the relevant PCA component scores were assessed with ICCs, calculated using mean rating (k = 2), two-way mixed effects models, with absolute agreement used for intra-rater reliability and consistency agreement used for inter-rater reliability (50).

Finally, all the measures were combined to create an overall stress measure using a factor analysis. Measures that occurred too infrequently to analyze during the individual analyses (>75% of observations scored as 0) were removed. Any missing data were imputed using multiple imputation. The data were then scaled and centered since the units for all measures were on different scales. A factor analysis with a varimax rotation was used to identify factors of interest, and the weightings from this analysis were used to generate factor scores. Factor scores were then modeled as above using linear mixed effects models, to determine the effect of treatment and timepoint. Separate analyses were run with all the data combined, and for the car and separation tests individually.

## Results

### Physiological measures of stress

#### Serum cortisol, immunoglobulin A, and glucose

**Figure 3:**
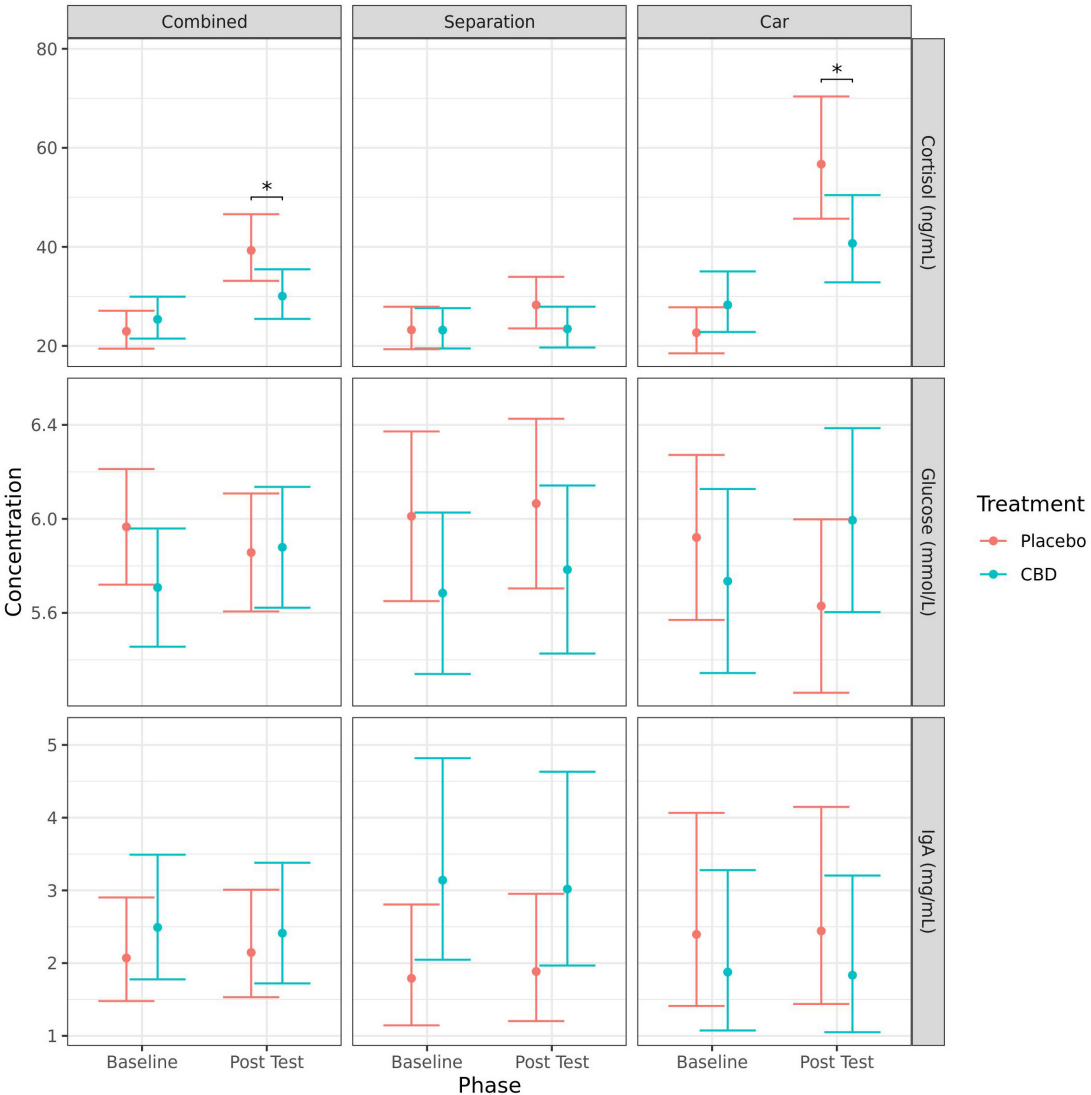
Predicted mean (95% CI) serum cortisol (ng/ml), glucose (mmol/L), and IgA (mg/ml) concentrations for dogs given CBD or placebo at each phase of testing (baseline and post-stressor) based on models analyzing both stress tests combined, separation test or car test. Asterisks indicate significant differences between treatment groups within each phase. *p < 0.05.

Due to a lack of homoscedasticity of the residuals, a log-transformation was applied to the models for cortisol and IgA. Considering changes from baseline to post-test, cortisol concentrations (mean ± SE) significantly increased for the placebo group when stress tests were combined (23.0 ± 1.9 vs. 39.3 ± 3.4 ng/ml, *p* < 0.001), and for the car test (22.7 ± 2.3 vs. 56.7 ± 6.0 ng/ml, *p* < 0.001) but not the separation test alone (23.2 ± 2.1 vs. 28.3 ± 2.6 ng/ml, *p* = 0.105). Cortisol also significantly increased for the CBD group for the car test (28.3 ± 3.0 vs. 40.7 ± 4.3 ng/ml, *p* = 0.028), but not for the separation test or when the stress tests were combined. There were no significant changes from baseline to post-test for IgA or glucose (Figure 3).

Overall, there were statistically significant differences between treatment groups based on serum cortisol concentrations, but not for IgA or glucose (Figure 3). Dogs given CBD had lower concentrations of serum cortisol post-test compared to dogs given placebo when the stress tests were analyzed together (30.0 ± 2.5 vs. 39.3 ± 3.4 ng/ml, *p* = 0.028) and for the car test when analyzed separately (40.7 ± 4.3 vs. 56.7 ± 6.0 ng/ml, *p* = 0.034). Although the mean post-test cortisol concentrations in the CBD group were numerically lower than the placebo group after the separation test, that difference was not statistically significant (23.4 ± 2.0 vs. 28.3 ± 2.6 ng/ml, *p* = 0.142). There was also a significantly greater increase in cortisol concentrations from baseline to post-test for the placebo group compared to the CBD group for both the combined stress tests (*p* = 0.027) and the car stress test (*p* = 0.017), but not the separation test (*p* = 0.257).

#### Eye, nose, and ear temperature

**Figure 4:**
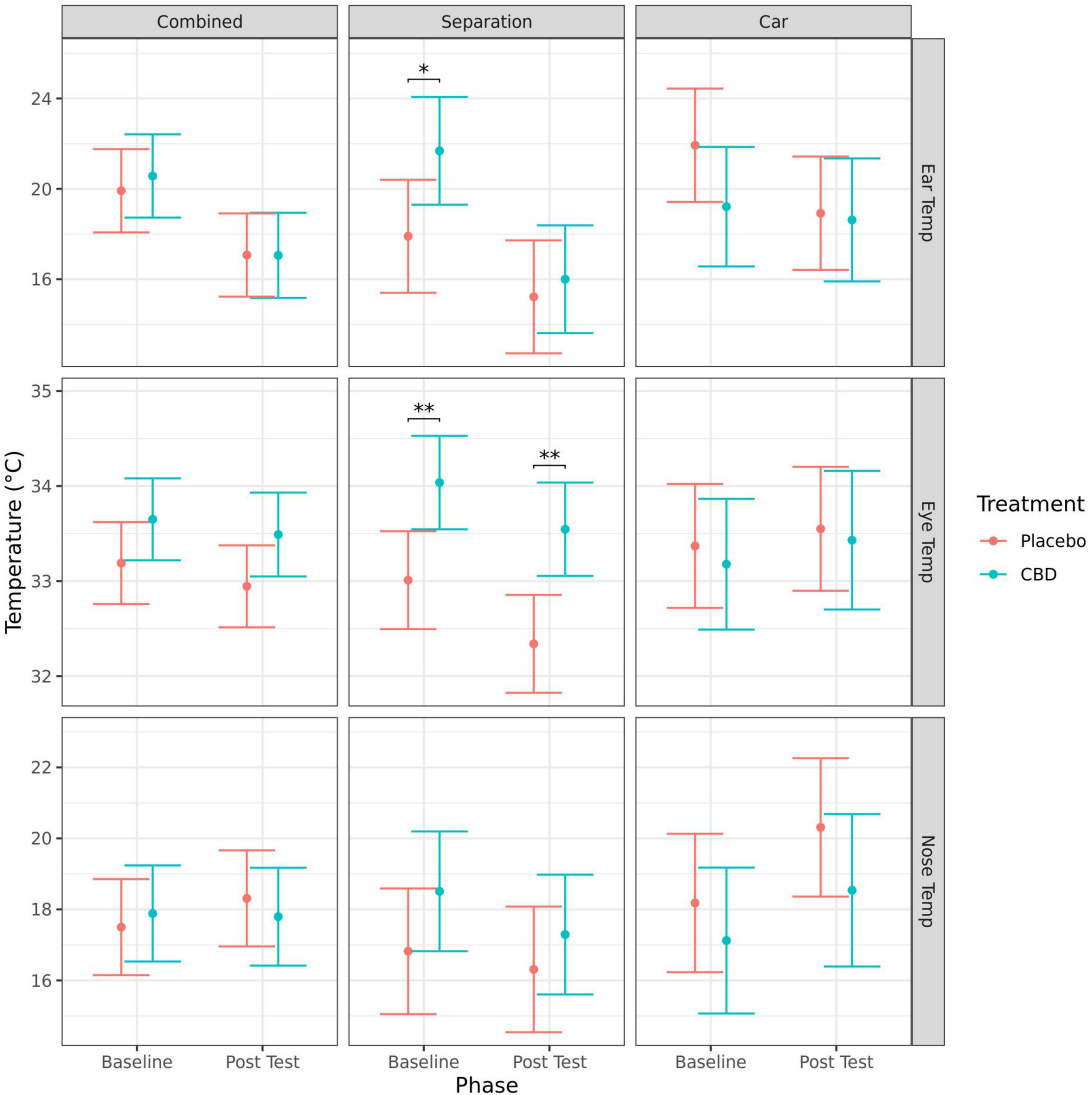
Predicted mean (95% CI) ear, eye and nose temperature (°C) measured using infra-red thermography for dogs given CBD or placebo at each phase of testing (baseline and post-stressor) based on models analyzing both stress tests combined, separation test or car test. Asterisks indicate significant differences between treatment groups within each phase. **p < 0.01, and *p < 0.05.

Mean ear temperature (mean ± SE) significantly decreased from baseline to post-test for both CBD (20.6 ± 0.9 vs. 17.1 ± 0.9°C, *p* = 0.002) and placebo (19.9 ± 0.9 vs. 17.1 ± 0.9°C, *p* = 0.009) group dogs when the tests were combined, for the CBD group in the separation test (21.7 ± 1.2 vs. 16.0 ± 1.2°C, *p* = 0.002), and for the placebo group in the car test (21.9 ± 1.2 vs. 18.9 ± 1.2°C, *p* = 0.005). There were no other significant effects of timepoint on infra-red temperature measurements (Figure 4).

Dogs in the CBD group had significantly higher mean ear temperatures compared to the placebo group at baseline (21.7 ± 1.2 vs. 17.9 ± 1.2°C, *p* = 0.033), and higher mean eye temperature at baseline (34.0 ± 0.2 vs. 33.0 ± 0.3°C, *p* = 0.006) and post-test (33.5 ± 0.2 vs. 32.3 ± 0.3°C, *p* = 0.002) following the separation test. There was no significant effect of treatment on the change of any of the temperature measurements from baseline to post-test (Figure 4).

#### Heart rate and heart rate variability

**Figure 5:**
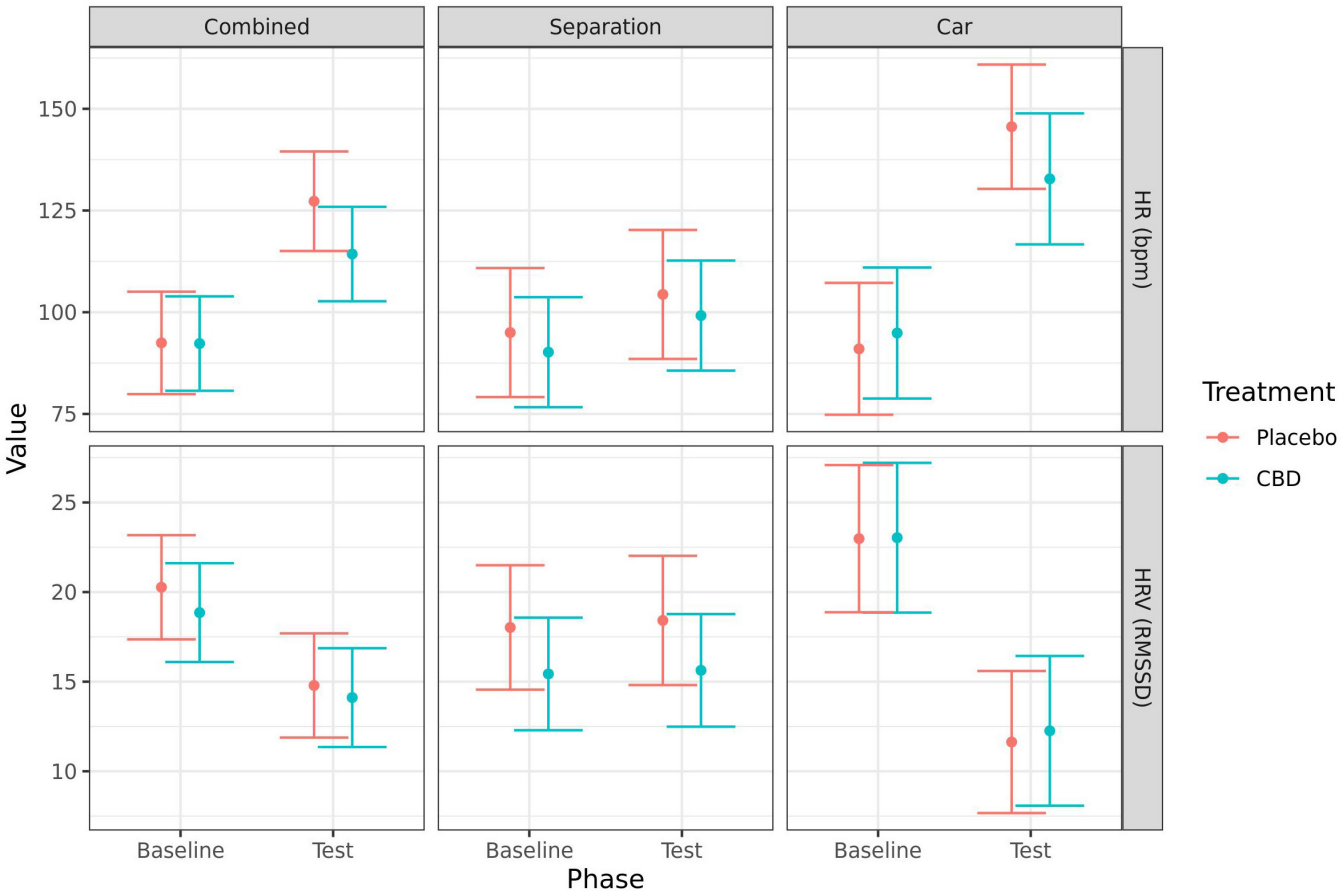
Predicted mean (95% CI) heart rate (beats/min) and HRV (RMSSD) for dogs given CBD or placebo at each phase of testing (baseline and test) based on models analyzing both stress tests combined, separation test or car test. No significant differences (p < 0.05) were identified between treatment groups within each phase.

HR (mean ± SE) significantly increased from baseline to post-test when the tests were combined (CBD: 92.3 ± 5.8 vs. 114.0 ± 5.8, *p* = 0.005; placebo: 92.5 ± 6.3 vs. 127 ± 6.1, *p* < 0.001) and for the car test alone (CBD: 94.9 ± 7.9 vs. 133.0 ± 7.9, *p* = 0.004; placebo: 91.0 ± 8.0 vs. 146.0 ± 7.5, *p* < 0.001). While there was a numerical increase in the mean HR for both groups in the separation test alone, this was not statistically significant (CBD: 90.2 ± 6.6 vs. 99.2 ± 6.6, *p* = 0.134; placebo: 95.0 ± 7.7 vs. 104.0 ± 7.7, *p* = 0.180). Additionally, HRV (mean RMSSD ± SE) significantly decreased from baseline to post-test when the tests were combined (CBD: 18.9 ± 1.4 vs. 14.1 ± 1.4, *p* = 0.009; placebo: 20.3 ± 1.5 vs. 14.8 ± 1.5, *p* = 0.005) and for the car test alone (CBD: 23.0 ± 2.0 vs. 12.3 ± 2.0, *p* < 0.001; placebo: 23.0 ± 2.0 vs. 11.6 ± 1.9, *p* < 0.001). However, there were no significant effects of treatment on HR or HRV at either timepoint, or in the change from baseline to post-test (Figure 5).

### Behavioral measures

#### Qualitative behavior assessment

Analysis of the QBA data using a PCA suggested one primary component of interest based on the strength of loadings and the variance explained (Table 3). The first component explained 56.4% of the total variance and was labeled PC1-Stressed/Anxious. It comprised of positive loadings for the terms “anxious,” “uncomfortable,” “restless,” “tense,” “stressed,” “nervous,” “reactive,” and “alert,” and negative loadings for the terms “calm,” “comfortable,” “relaxed,” and “lethargic,” “Fearful,” and “nauseous” did not occur frequently enough to analyze (>75% of occurrences scored as 0) therefore these terms were not included in the PCA and the corresponding results are not presented.

**Table 3:** Components extracted by the PCA of QBA terms.

**Term**	**PC1**	**PC2**	**PC3**	**PC4**
Uncomfortable	**0.92**	0.24	0.15	0.10
Anxious	**0.90**	0.10	0.11	0.16
Restless	**0.89**	−0.02	0.05	0.06
Tense	**0.89**	0.20	0.22	0.02
Stressed	**0.88**	0.18	0.24	0.18
Nervous	**0.85**	−0.01	0.33	0.17
Reactive	**0.74**	−0.40	−0.10	−0.13
Alert	**0.65**	−0.39	−0.23	−0.40
Comfortable	**−0.89**	−0.09	0.19	0.10
Calm	**−0.87**	0.16	−0.04	−0.01
Relaxed	**−0.86**	0.01	0.20	0.13
Lethargic	**−0.56**	0.47	0.42	0.17
Depressed	0.03	**0.86**	−0.32	−0.08
Sad	0.33	**0.77**	−0.39	−0.11
Explorative	0.09	−0.26	**−0.55**	**0.78**
Variance Explained (%)	56.2	14.0	7.5	6.4

Loadings ≥0.50 are in bold.

Inter-rater reliability for the individual QBA terms indicated agreement was poor to good, with poor reliability (ICC < 0.50) for the term “relaxed,” moderate reliability (ICC 0.50-0.75) for the terms “alert,” “anxious,” “calm,” “comfortable,” “depressed,” “explorative,” “reactive,” “restless,” “sad,” and “tense,” and good reliability (ICC 0.75-0.90) for the terms “lethargic,” “nervous,” “stressed,” and “uncomfortable.” Inter-rater reliability for the PC1-Stressed/Anxious component score was good (ICC = 0.83).

Intra-rater reliability for the individual QBA terms indicated agreement was poor to excellent depending on the term and rater. The term “lethargic” was not analyzed as it occurred too infrequently in the selected videos. For rater 1, reliability was poor for the terms “alert,” “depressed,” and “sad,” moderate for the terms “restless” and “tense,” good for the terms “anxious,” “explorative,” “nervous,” “reactive,” “relaxed,” “stressed,” and “uncomfortable” and excellent for the terms “calm” and “comfortable.” For rater 2, reliability was moderate for the terms “alert,” “explorative,” “restless,” “stressed,” and “tense,” good for the terms “anxious,” “calm,” “comfortable,” “nervous,” “reactive,” “relaxed,” “sad,” and “uncomfortable” and excellent for the term “depressed.” Intra-rater reliability for the PC1-Stressed/Anxious component score was excellent for rater 1 (ICC = 0.92) and good for rater 2 (ICC = 0.85).

When comparing baseline to during test, dogs scored significantly higher during the test on the QBA PC1—Stressed/Anxious component score regardless of treatment when analyzed combined (CBD: *p* < 0.001; placebo: *p* < 0.001), for the separation test (CBD: *p* = 0.013; placebo: *p* = 0.014), and the car test (CBD: *p* < 0.001; placebo: *p* < 0.001). Detailed results for the analyses of individual terms from the QBA are presented in the Supplementary material. In summary, dogs in both treatment groups scored as significantly (*p* < 0.050) more “anxious,” “explorative,” “stressed,” and “uncomfortable” during the separation test, and less “relaxed” when compared to baseline. Only dogs in the placebo group scored as more “nervous,” “sad,” and “tense” during the separation test compared to baseline, while only dogs in the CBD group scored as less “comfortable” and more “restless” during the separation test compared to baseline. During the car test, dogs in both treatment groups scored as significantly more “anxious,” “nervous,” “restless,” “stressed,” “tense,” “uncomfortable,” and less “calm” when compared to baseline. Only dogs in the placebo group scored as less “comfortable” and “relaxed” during the car test compared to baseline, while only dogs in the CBD group scored as more “explorative” during the car test compared to baseline. There were no significant effects of time point on the terms “alert,” “depressed,” or “reactive.”

When analyzed combined, dogs in the CBD group scored as significantly less “sad” (*p* = 0.004) and had a tendency to be more “explorative” (*p* = 0.080) during the tests when compared to the placebo group. Dogs in the CBD group also had a tendency for a greater increase in “explorative” ratings (*p* = 0.091), and smaller increase in “sad” ratings (*p* = 0.051) from baseline to test when compared to the placebo group.

For the separation test, dogs in CBD group scored as significantly less “sad” (*p* = 0.032), less “stressed” (*p* = 0.014), less “tense” (*p* = 0.011), and less “uncomfortable” (*p* = 0.028) during the test when compared to dogs given placebo. There was a tendency for dogs in the CBD group to have a greater increase in “explorative” ratings (*p* = 0.097) from baseline to separation test compared to the placebo group.

**Figure 6:**
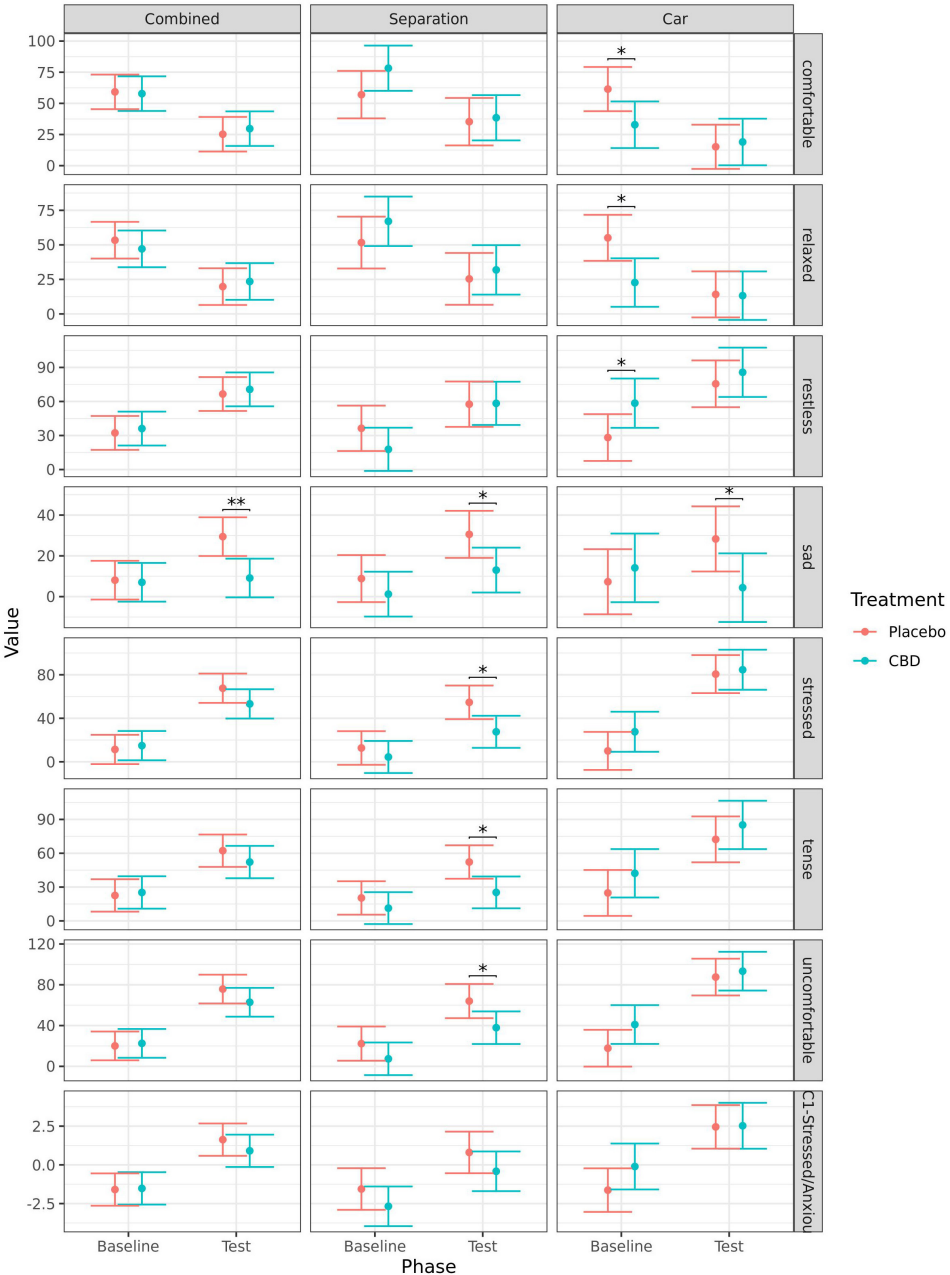
Predicted mean (95% CI) QBA ratings for the terms “comfortable,” “relaxed,” “restless,” “sad,” “stressed,” “tense,” “uncomfortable,” and the component score for PC1-Stressed/Anxious for dogs given CBD or placebo at each phase of testing (baseline and test) based on models analyzing both stress tests combined, separation test or car test. Asterisks indicate significant differences between treatment groups within each phase. **p < 0.01, and *p < 0.05.

For the car test, dogs in the CBD group scored as significantly less “comfortable” (*p* = 0.031), less “relaxed” (*p* = 0.010), and more “restless” (*p* = 0.048) at baseline, but this difference was no longer present during the test. During the car test, dogs in the CBD group scored as significantly less “sad” (*p* = 0.044). Dogs in the CBD group had a significantly smaller decrease in “relaxed” ratings (*p* = 0.033) from baseline to the car test, and a tendency for a smaller decrease in “calm” (*p* = 0.051) and “comfortable” (*p* = 0.062) ratings, and a smaller increase in “sad” ratings (*p* = 0.071) compared to dogs in the placebo group. There were no other significant effects of treatment (Figure 6).

#### Coded behaviors

Due to a high incidence of zero occurrence for several behaviors, analyses were only conducted for behaviors with <75% zero values. This resulted in only whining being analyzed from the combined analysis and separation test, and only lip licking being analyzed from the car test.

**Figure 7:**
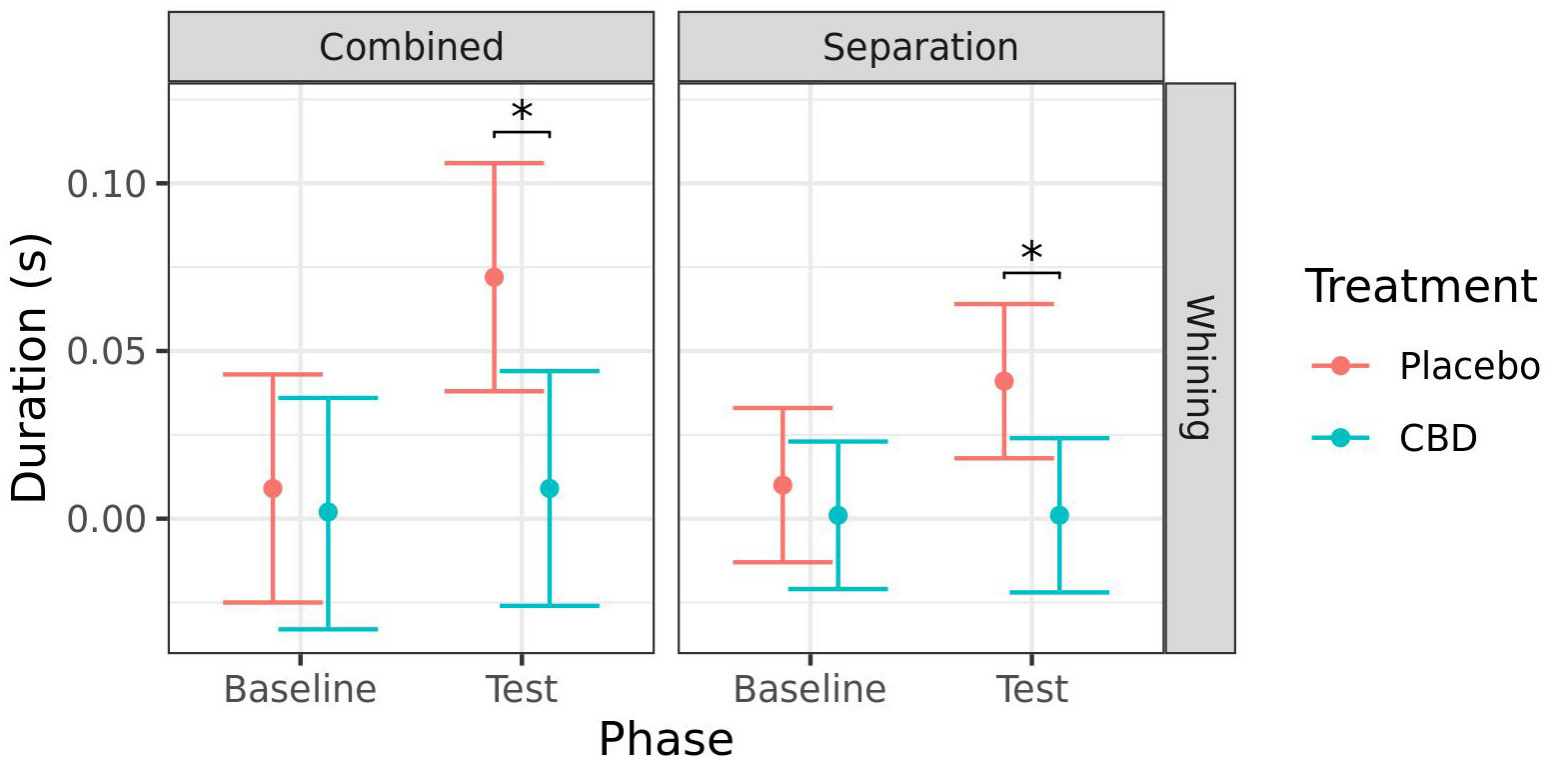
Predicted mean (95% CI) duration of time spent whining(s) for dogs given CBD or placebo at each phase of testing (baseline and test) based on models analyzing both stress tests combined or separation test. Asterisks indicate significant differences between treatment groups within each phase. *p < 0.05.

There was no significant effect of timepoint on whining in the CBD group for either test (combined: *p* = 0.761; separation: *p* = 0.993). Whining was significantly lower during the test session for the CBD group compared to the placebo group when analyzed combined (*p* = 0.013), and for the separation test (*p* = 0.019). There was also a significant increase in whining from baseline to test in the placebo group when analyzed combined (*p* = 0.013) but the increase for the separation test was not quite significant (*p* = 0.067; Figure 7).

**Figure 8:**
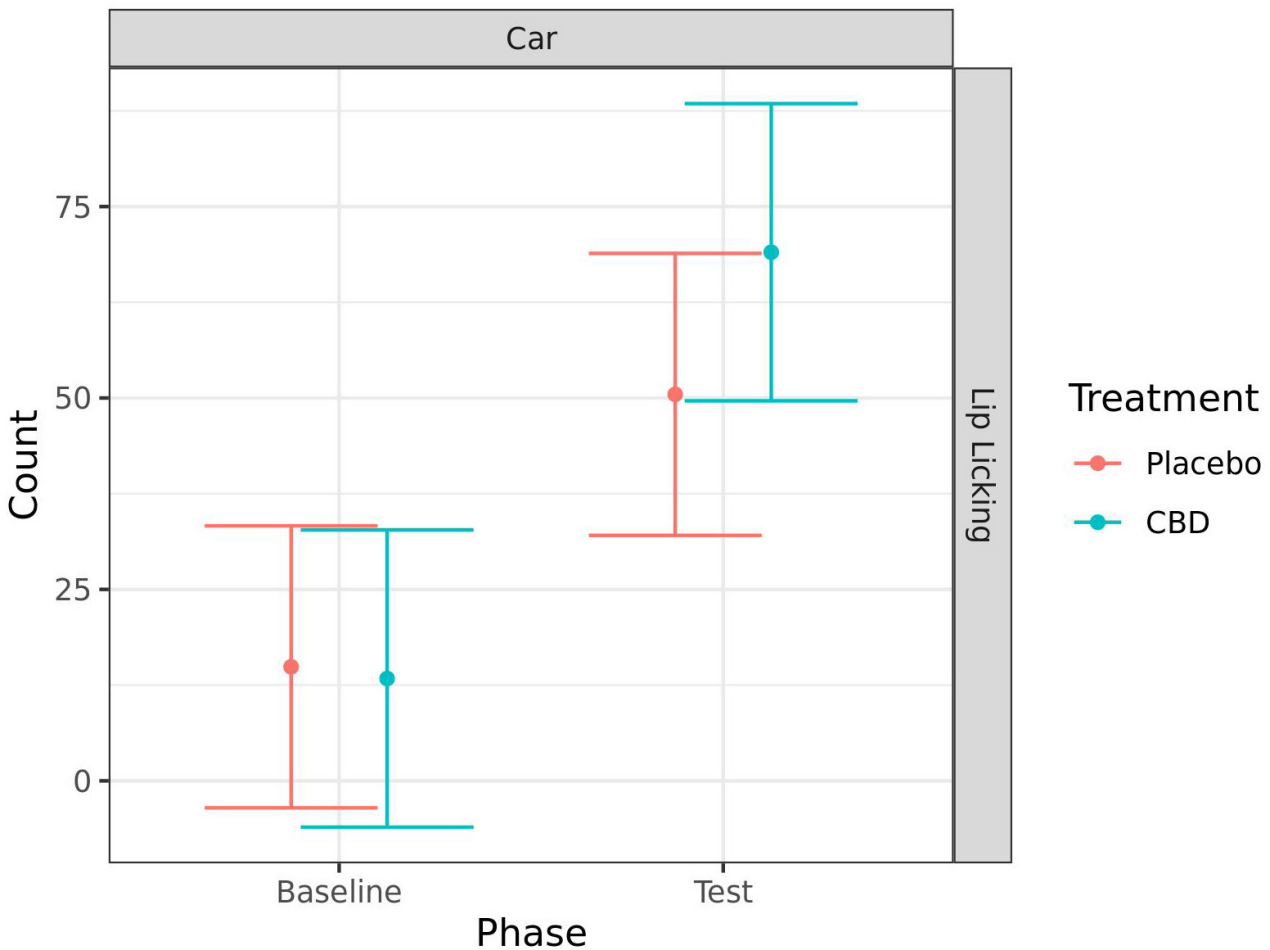
Predicted mean (95% CI) number of lip licks for dogs given CBD or placebo at each phase of testing (baseline and test) during the car test. No significant differences (p < 0.05) were identified between treatment groups within each phase.

Lip licking significantly increased from baseline to during the car test for both treatment groups (CBD: *p* < 0.001; placebo: *p* = 0.005). However, there was no significant effect of treatment on this behavior (Figure 8).

#### Body position and activity

**Figure 9:**
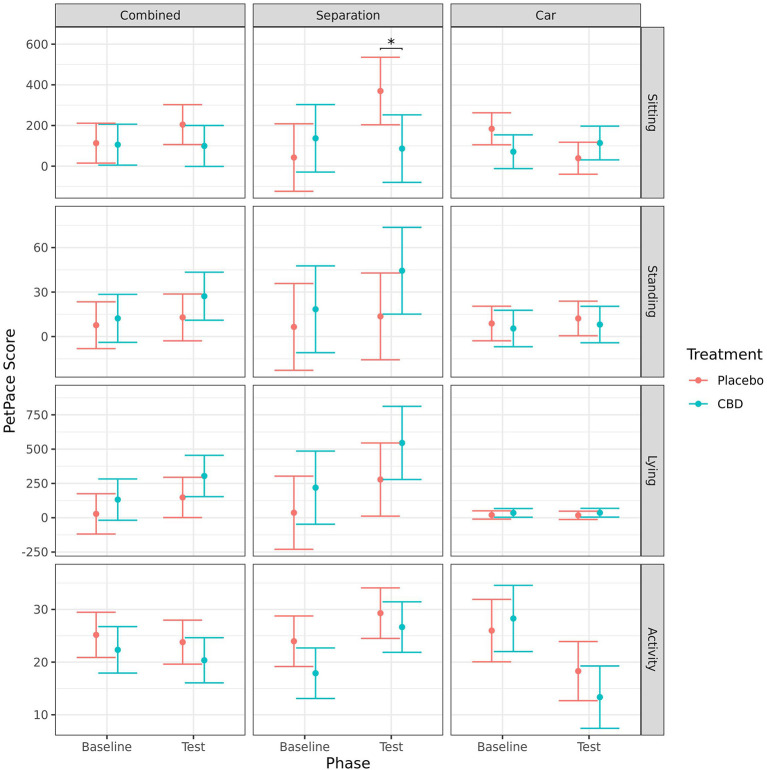
Predicted mean (95% CI) “sitting,” “standing,” “lying,” and “activity” scores generated by the PetPace™ device for dogs given CBD or placebo at each phase of testing (baseline and test) based on models analyzing both stress tests combined, separation test or car test. Asterisks indicate significant differences between treatment groups within each phase. *p < 0.05.

Dogs in the placebo group had a significant increase in sitting from baseline to test for the separation test (*p* = 0.011), but a decrease in sitting for the car test (*p* = 0.004). Consequently, there was no effect of timepoint when analyzed combined, and there was no effect of timepoint on the CBD group for any of the tests. Dogs in the placebo group spent significantly more time sitting during the separation test (*p* = 0.019) but showed no significant difference in the car test (*p* = 0.191). The change in sitting behavior from baseline to post-test was significantly greater for the placebo group than the CBD group in both the separation test (*p* = 0.033) and car test (*p* = 0.009). When both tests were analyzed together there were no significant effects of treatment on sitting. There were no significant effects of treatment or timepoint during either of the stress tests on amount of standing or lying as measured using the PetPace™ device (Figure 9).

There were no significant effects of treatment or timepoint on activity when the stress tests were analyzed combined. For the separation test, there was a significant increase in activity from baseline to during test for dogs in the CBD group (*p* = 0.017). This is at least partly due to the tendency for activity to be lower at baseline for dogs in the CBD group compared to dogs in the placebo group (*p* = 0.078). However, the change in activity from baseline to during test was not significantly different between the treatment groups (*p* = 0.478). For the car test, there was a significant decrease in activity from baseline to during test for both treatment groups (CBD: *p* < 0.001; placebo: *p* = 0.042), but there was no significant effect of treatment (Figure 9).

#### Distance traveled

**Figure 10:**
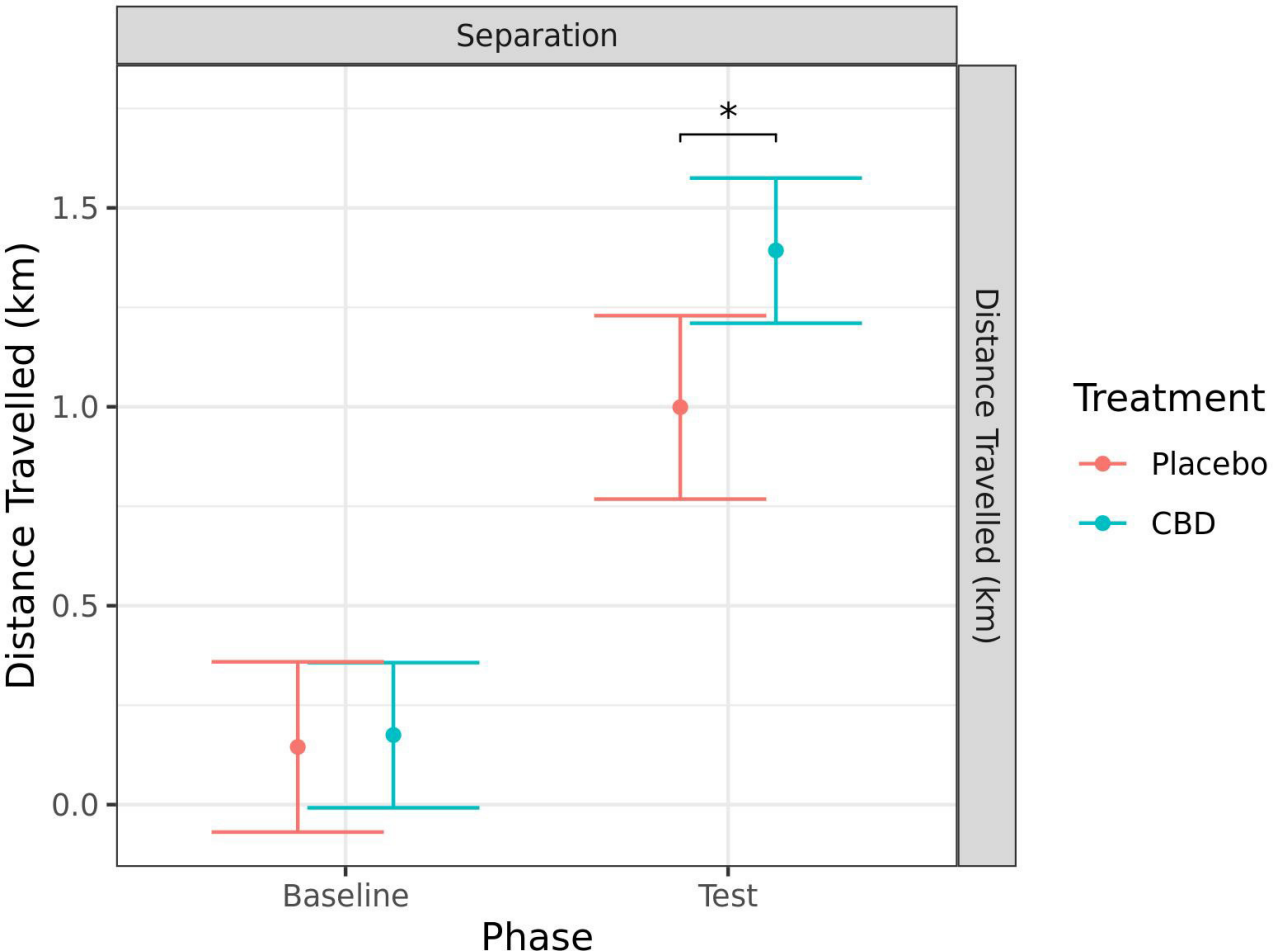
Predicted mean (95% CI) distance traveled (km) generated by the Polar^®^ device for dogs given CBD or placebo at each phase of testing (baseline and test) during the separation test. Asterisks indicate significant differences between treatment groups within each phase. *p < 0.05.

Distance traveled was measured using the Polar^®^ device for the separation test only. There was a significant increase in distance traveled from baseline to during separation for both the CBD (*p* < 0.001) and placebo (*p* < 0.001) treatment groups. In addition, there was a significant difference between treatment groups in the distance traveled during the test, with dogs given CBD traveling further (*p* = 0.010). There was a tendency for dogs in the CBD group to have a greater increase in distance traveled from baseline to during test when compared to the placebo group (*p* = 0.086; Figure 10).

### Combined stress measure

After removal of measures with low levels of occurrence, the following measures were included in the combined analysis: cortisol, IgA, glucose, mean ear temp, mean eye temp, mean nose temp, HR, HRV, QBA PC1-Stressed/Anxious component, sitting, lying, standing, and activity. In addition, lip licking was included in the car analysis, distance traveled was included in the separation analysis, and whining was included in the combined and separation analysis. Results of the factor analyses suggested two factors that were common across the car, separation and combined analyses based on the strength of loadings and the variance explained (Table 4). The first factor (MR1) was labeled “stressed” and explained 17.6–24.7% of the total variance depending on the data included and was used as the combined stress measure. Loadings for individual measures varied depending on the data included, but for all the analyses the first factor included positive loadings for cortisol, HR, and the QBA PC1-Stressed/Anxious component. Additionally, there were positive loadings for activity, whining and distance traveled in the separation analysis, and positive loadings for lip licking, and negative loadings for activity and HRV in the car analysis.

**Table 4:** Factors extracted by the factor analysis of all measures when analyzed with both stress tests combined, or with separation and car tests separately.

	**Combined**		**Separation**		**Car**	
**Measure**	**MR1 stress**	**MR2 temp**	**MR1 stress**	**MR2 temp**	**MR1 stress**	**MR2 temp**
HR	**0.86**	0.06	**0.58**	0.01	**0.80**	0.10
QBA PC1	**0.84**	−0.04	**0.90**	−0.18	**0.74**	0.26
Cortisol	**0.73**	0.22	**0.52**	0.07	**0.71**	0.26
Activity	−0.06	−0.32	**0.68**	−0.17	**−0.69**	−0.13
Whining	0.43	0.01	**0.56**	0.12	**–**	**–**
Distance traveled	**–**	-	**0.53**	−0.34	**–**	**–**
Lip licking	–	**–**	**–**	–	**0.68**	0.01
HRV	−0.38	−0.17	−0.04	−0.31	**−0.81**	0.05
Nose mean	0.27	**0.80**	0.00	**0.73**	0.26	**0.85**
Ear mean	−0.03	**0.73**	−0.20	**0.86**	−0.07	**0.72**
Eye mean	−0.09	**0.68**	−0.29	**0.50**	0.08	**0.69**
Standing	−0.03	0.04	0.08	−0.04	−0.01	−0.08
Sitting	−0.14	0.05	0.03	0.09	−0.07	−0.07
Lying	−0.15	0.04	0.02	−0.06	−0.14	−0.01
Glucose	0.13	−0.12	0.15	−0.19	0.16	0.04
IgA	−0.36	0.01	−0.20	0.11	−0.26	−0.38
Variance explained (%)	18.4	13.2	17.6	12.4	24.7	14.4

Loadings ≥0.50 are in bold.

One additional factor was identified (MR2) and was labeled “temperature” which explained 12.4–14.4% of the total variance depending on the data included. This “temperature” factor included positive loadings for mean nose, eye, and ear temperatures for all the analyses. The only measures that did not load on any factor for any of the analyses were standing, sitting, lying, blood glucose, and IgA.

**Figure 11:**
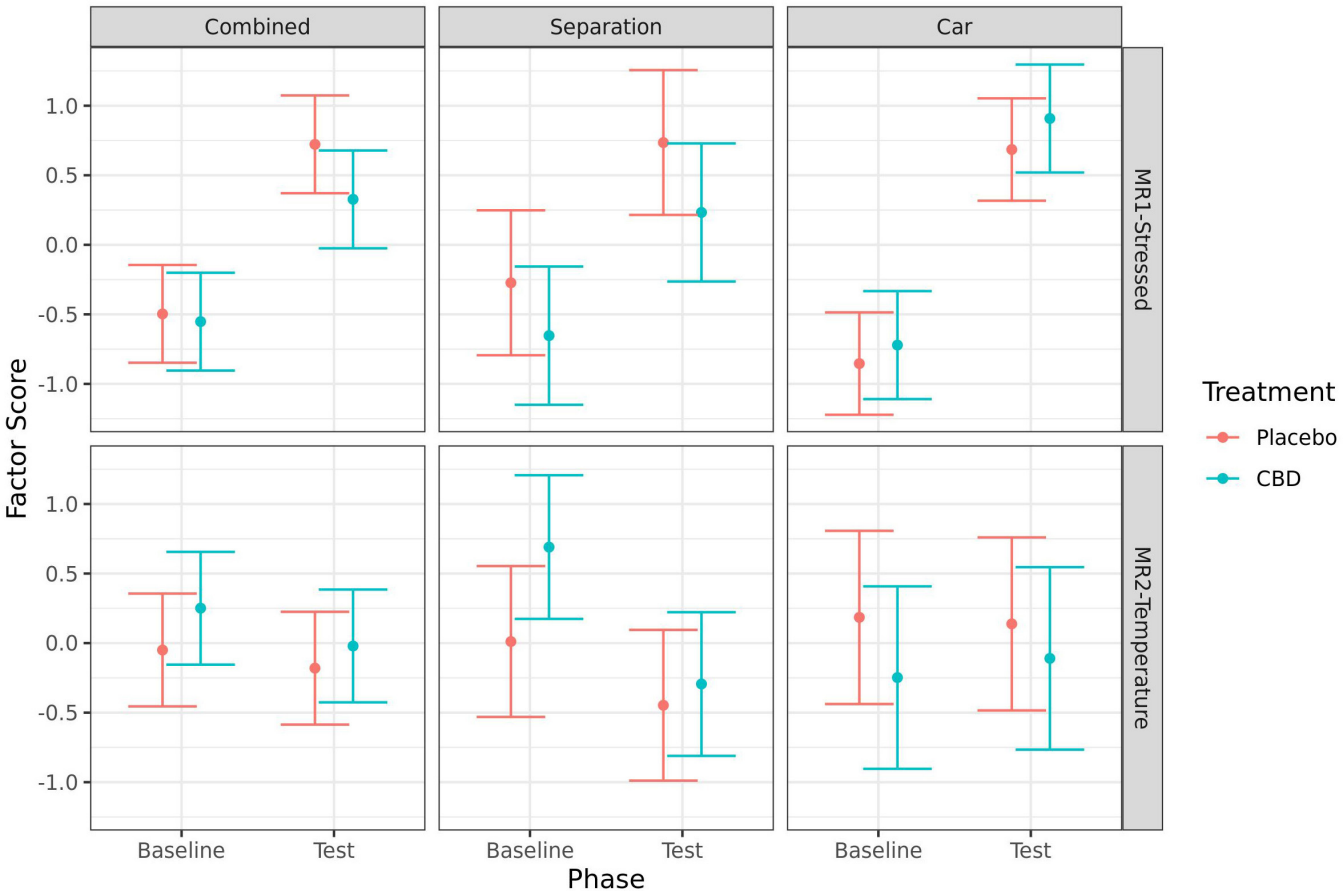
Predicted mean (95% CI) factor scores indicating levels of “stressed” (MR1), or “temperature” (MR2) for dogs given CBD or placebo at each phase of testing (baseline and test) based on models analyzing both stress tests combined, separation test or car test. Asterisks indicate significant differences between treatment groups within each phase. No significant differences (p < 0.05) were identified between treatment groups within each phase.

Results of the analyses of the first factor indicate that dogs were more “stressed” at the test timepoint compared to baseline for both the CBD group (combined: *p* < 0.001; separation: *p* = 0.012; car: *p* < 0.001), and the placebo group (combined: *p* < 0.001; separation: *p* = 0.007; car: *p* < 0.001). There were no significant effects of treatment on the combined stress measure at either timepoint, or on the change from baseline to test. The second factor indicated dogs in the CBD group were less “hot” during the separation test when compared to baseline (*p* = 0.008), but not in the placebo group (*p* = 0.207). There were no other significant effects of timepoint or treatment on the “temperature” factor (Figure 11).

## Discussion

Despite relatively little empirical evidence supporting the use of CBD to alleviate acute or chronic stress in dogs, CBD is increasingly being discussed as a potential intervention in veterinary consultations (51). Further, it is widely available in a range of products claiming positive benefits, stress-reducing properties and improved pet emotional wellbeing. The aims of this study were to understand the impact a period of separation and car travel had on measures of stress in dogs, and evaluate the effect a single dose of CBD at 4 mg/kg had on these measures. Overall, it was evident that some measures of canine stress changed significantly from baseline after exposure to these specific events, suggesting the situations elicited a negative emotional response in dogs. Additionally, some measures of stress in dogs were significantly affected following administration of CBD, suggesting it may have efficacy as an intervention for acute stress in dogs.

### Exposure to a separation event and car travel

Pet dogs routinely experience being left alone, as well as traveling in a vehicle, although the absolute and relative impacts that these events have on both physiological and behavioral measures of canine stress are not well-understood. In this study a more notable effect was observed between various baseline measures and exposure to car travel, compared to baseline and separation test. This suggests traveling in a vehicle was likely more stressful than being separated from conspecifics and humans. Unlike many pet dogs, this particular population do not routinely undergo car travel, so the novelty of the event may have caused a more pronounced stress response. However, the results are consistent with other studies that reported car travel elicits stress in dogs, demonstrated by an increase in salivary or plasma cortisol (9, 52) and restless behavior (53). Dogs can suffer from motion sickness, which may also contribute to the elevation of stress (3, 22).

Typically, information related to how dogs cope with car travel is documented *via* owner-reported surveys (4), whereas here behavioral and physiological parameters were captured. Serum cortisol and heart rate, both widely used physiological measures of mammalian stress, were elevated significantly following exposure to the car test. The elevated cortisol levels observed in the current study were similar to those recorded in dogs in response to stress in a veterinary clinic (54, 55). The hypothalamic-pituitary-adrenal (HPA) axis is activated during a stress response with the sympathetic nervous system responsible for releasing adrenaline and noradrenaline (56). These hormones increase HR and lower HRV, while also increasing respiration. Although they are commonly used measures in animal welfare science, factors such as circadian rhythms and exercise can impact cortisol and HR independent of stress (57). However, this study was designed to control these factors with feeding, exercise, husbandry activities and sampling all conducted at the same time each day within a colony environment. Additionally, behavioral parameters such as lip licking, relevant QBA terms, the QBA PC1-Stressed/Anxious component score and the combined stress measure also increased, with heart rate variability and activity significantly decreasing. Consistent with these observations, studies examining canine stress have described cortisol levels increasing (58, 59) and HRV lowering in response to a perceived negative stimulus (60, 61), with dog behaviors related to lip licking, such as hypersalivation, barking and restlessness occurring more frequently during car travel (4). While the reduction in recorded activity was likely caused by being enclosed in a crate during the car journey, it also confirms that elevated HR was not due to increased physical activity, but instead a direct response to the stressor. When these combinations of measures are considered together, the car test appears to elicit a range of both behavioral and physiological measures of stress.

During the separation test, the physiological parameters were not significantly affected, however the combined stress measure labeled “stressed” significantly increased from baseline. In addition, the dogs were rated higher on the QBA PC1-Stressed/Anxious component and as being more “anxious,” “explorative,” “nervous,” “sad,” “stressed,” “tense” and “uncomfortable,” and less “relaxed.” Dogs were indeed significantly more active, traveling greater distances during the separation test compared to baseline. This may be because baseline recordings were recorded in their home pen, a familiar environment that is, on average a third smaller than the separation test room, potentially resulting in less exploratory behavior. However, increased motor activity has been observed in dogs suffering from separation-related anxiety previously (6). Dogs also sat significantly more during the separation test. When considered with the decreased ratings for “relaxed,” this suggests that stationary behavior may not always be interpreted as relaxation. Indeed, the dogs often sat by the door, possibly awaiting their caregiver's return. This type of behavior, coupled with increased activity and arousal, may be related to hypervigilance which is commonly observed in dogs (62), cats (63), and rats (64) experiencing a stress response. In extreme cases, stress can result in immobility as active stress becomes passive, a behavior more commonly reported in rodents and rabbits (65, 66).

Whining also occurred significantly more during the separation test compared to the baseline timepoint. This behavior has also been reported previously, with increases in vocalization when an owner leaves their dog (67, 68). A limitation of the present study is that the dogs involved are not “owned” although they do form attachments with staff, and during this study a familiar caregiver left them in the test space. The dogs are rarely socially isolated from humans or conspecifics. Therefore, it is likely this population of dogs experienced mild stress in response to being both separated from their caregiver, as well as being socially isolated (69). It has been demonstrated that hyper-attachment greatly increases not only the incidence of separation-related anxiety, but also increases the severity (25). Therefore, while some markers of stress were apparent, this separation paradigm may not be fully reflective of the experience a pet dog experiences when their owner leaves them at home.

Not all measures were significantly different between baseline and test sessions and the direction of some differences may be context dependent. Eye, ear and nose temperatures captured using infrared thermography have previously been used for evaluating stress in dogs (46, 70), though there are inconsistencies as to whether an increase or decrease in temperature is indicative of stress. Stress-induced hyperthermia is known to occur in response to a short-term stressor in dogs (71) and other mammalian species (72, 73), with thermogenic readings of the eyelid and lacrimal caruncle, providing a non-invasive approach to measure the phenomenon (47, 71). Dog ear temperature was found to decrease in a previous study evaluating the effect of periods of separation (74), which is comparable to findings here. In contrast, dog eye temperature was observed to increase when dogs were in a veterinary setting (46, 70) which was also interpreted as a stress-related response. The variability in body temperature may be influenced by environmental factors in the different test settings. Local temperature and relative humidity were not captured during this study. This is a limitation as these factors may have allowed environmental influences on the change in surface body temperature to be distinguished from physiological responses indicative of stress. While baseline and test sessions were held in a climate-controlled environment, prior to the post-stress measure dogs were walked outside briefly to access the sample room. Further, the decrease in temperature observed in this study was only significant for the ear temperature measure. As this was a measure of the fur covered external surface of the ear pinnae, it is likely it was more heavily influenced by external temperature, and less directly related to the dogs' surface temperature when compared to the eye and nose measurements. It is therefore possible that surface body temperature may not be suitable as a standardized measure of canine stress unless under highly controlled conditions.

Overall, these data provide a more detailed understanding on how dogs cope during these routine events. The results also further highlight the importance of a multiple measures approach to better understand the impact certain scenarios have on canine wellbeing, a routinely recommended approach in animal welfare studies (46, 75, 76). Gathering a range of data from dogs during these situations and identifying appropriate ways to measure canine wellbeing is a necessary step prior to evaluating the effectiveness of interventions aimed at alleviating canine stress. It is evident that measures are not uniform across the scenarios, suggesting certain parameters may be more, or less, applicable in different testing situations.

### Effect of CBD on measures of canine stress

The study determined that a single 4 mg/kg dose of CBD distillate influences some behavioral and physiological parameters in dogs following exposure to the two different stress-inducing events. During car travel, CBD treated dogs were scored as significantly less “sad,” and also had a smaller decrease in “relaxed” ratings from baseline to test when compared to the placebo treated dogs. Dogs that received the CBD treatment also had significantly lower serum cortisol concentrations than dogs that received a placebo. CBD contributes to lowering cortisol levels, possibly by regulation of the HPA axis *via* inhibition of fatty acid amide hydrolase (FAAH) (77). In contrast, a modulatory effect was not observed for serum IgA or glucose concentrations between treatment and control groups. However, these measures also didn't change from baseline to post-test, suggesting they may not be appropriate measures of stress in these paradigms. There were no significant differences in HR or HRV between treatment and placebo groups. Administration of CBD has been reported to increase HR in dogs following exposure to fireworks sounds (43). Further, an increase in HR has been observed in humans and rats with no exposure to a stressor, potentially due to CBD attenuating the increase in blood pressure associated with stress, resulting in an increase in heart rate (78, 79). Therefore, it is perhaps unsurprising that the increase in HR was not buffered by CBD administration. When considered collectively, these results suggest that a single dose of CBD has a positive effect on reducing multiple aspects of canine stress during a car journey.

Dogs who received CBD were rated as being significantly less “stressed,” “sad,” “tense,” and “uncomfortable” and more “explorative” during the separation event than dogs who received the placebo. Consistent with this, dogs who received the CBD also exhibited less whining and sitting behavior and traveled further when they were left alone. Collectively these characteristics are suggestive of a more relaxed emotional state in CBD treated dogs. In other species, acute stress leads to an increase in activity/exploration, whereas chronic stress results in reduced exploration (80, 81). Further work will be required to fully understand the relationship between stress and activity in canines, and the impact CBD has on it. A previous study examined the impact CBD had specifically on aggressive dogs in a shelter environment, a behavior which can occur due to stress. Reduction in aggressive behavior was observed in the treatment group who were administered an unspecified dose of CBD over a period of 45 days (82). A more recent study investigated the efficacy a lower (1.4 mg/kg) CBD dose had on dogs who were exposed to firework-related sounds. A single CBD treatment showed no impact on plasma cortisol, HR and a range of behaviors associated with canine stress (43) however treats were administered 4–6 h prior to testing. Dogs in the present study received a larger oral dose of CBD *via* capsules that was administered more acutely, just 2 h prior to testing. Moreover, the molecular complexity of bioactives in different hemp distillates (potentially resulting in altered CBD bioavailability and pharmacokinetics) means that caution should be exercised before generalizing the results from this study to CBD-containing products more broadly.

Comparing the data from both stress events suggests that a single 4 mg/kg dose of CBD may be generally effective in alleviating acute stress responses in dogs. But different measures of canine stress appear to be more or less sensitive to CBD in different stressful scenarios. Some limitations of the study may contribute to this observation. No power analyses were conducted prior to the start of the study, as the sample size was instead determined by the parallel safety study (40). This likely resulted in insufficient power to detect significant differences in some measures. The study consisted of a parallel design, so individual differences in dog behavior and preferences may have contributed additional variation. It is possible more significant differences would be identified if a cross-over design was used. Kinetic analysis of CBD metabolism was not conducted as part of the present study. Based on some pharmacokinetic parameters, it is possible that the 2 h window between dosing and the stress event were not enough to observe the therapeutic effects in the dogs tested (38, 83). Only three dog breeds were represented, all clinically healthy and living in a homogeneous environment, and they were not “owned” pets selected for displaying separation-related anxiety or travel-related stress. Therefore, confirming these research findings in pet dogs in traditional home environments will be beneficial. Doses >4 mg/kg, have caused mild side effects in tolerance studies (39), but testing the efficacy of lower or multiple dosing of CBD in the same stress paradigms may also be worthwhile.

## Conclusions

The results obtained from this study suggest that a period of separation and car travel are stressful events for dogs, with travel in a vehicle eliciting a more pronounced stress response. Further, a single dose of 4 mg/kg CBD 2 h prior to exposure to these events attenuates some indicators of acute canine stress, which is likely to improve canine emotional wellbeing. Additional research is required to better understand the effect of CBD at other dosages, formulations, and whether cumulative administration improves efficacy.

## Data availability statement

The raw data supporting the conclusions of this article will be made available by the authors, without undue reservation.

## Ethics statement

This study was approved by the Waltham Animal Welfare and Ethical Review Body (80265) and conducted under the authority of the Animals (Scientific Procedures) Act 1986.

## Author contributions

AH, DL, and TK: conceptualization and design. AH and TK: methodology. HF: statistical analysis. AH: investigation and writing—original draft preparation. AH, HF, DL, and TK: writing—review and editing. All authors have read and agreed to the published version of the manuscript.
