# Supplementary material for: A single dose of cannabidiol (CBD) positively influences measures of stress in dogs during separation and car travel
Source: Front Vet Sci. 2023 Feb 22;10:1112604. doi: 10.3389/fvets.2023.1112604 (PMC9992179; doi:10.3389/fvets.2023.1112604)
Supplement: Supplementary file 1 [file Table_1.docx]

**Supplementary material**: Pairwise comparisons from linear mixed effects models of individual terms from the Qualitative Behaviour Assessment (QBA) looking at the difference in scores between baseline (BL) and test (T) timepoints, between CBD and placebo (PBO) treatment groups, and the interaction between timepoint and treatment. Separate analyses were run for dogs that experienced a separation test, car test, and combining results from both tests. A tukey adjustment has been applied to p-values to account for multiple pairwise comparisons.

| Term | Contrast | Analysis | | | | | | | | |
| --- | --- | --- | --- | --- | --- | --- | --- | --- | --- | --- |
|  |  | Combined | | | Separation | | | Car | | |
|  |  | Estimated Difference | SE | P-value | Estimated Difference | SE | P-value | Estimated Difference | SE | P-value |
| Alert | BL-T (CBD) | -9.6 | 7.1 | 0.187 | -4.2 | 10.2 | 0.683 | -16.1 | 10.3 | 0.136 |
|  | BL-T (PBO) | -10.8 | 7.1 | 0.138 | -10.9 | 10.7 | 0.319 | -10.7 | 9.7 | 0.289 |
|  | CBD -PBO (BL) | -3.2 | 8.0 | 0.694 | -2.6 | 11.0 | 0.812 | -3.0 | 11.7 | 0.8 |
|  | CBD - PBO (T) | -4.4 | 8.0 | 0.584 | -9.3 | 11.0 | 0.401 | 2.4 | 11.7 | 0.838 |
|  | (BL – T CBD) – (BL – T PBO) | 1.2 | 10.1 | 0.903 | 6.7 | 14.8 | 0.655 | -5.4 | 14.1 | 0.707 |
| Anxious | BL-T (CBD) | -36.3 | 7.0 | <0.001 | -33.3 | 9.6 | 0.003 | -39.9 | 10.1 | 0.001 |
|  | BL-T (PBO) | -44.9 | 7.0 | <0.001 | -35.4 | 10.1 | 0.002 | -54.3 | 9.6 | <0.001 |
|  | CBD -PBO (BL) | -0.1 | 9.0 | 0.996 | -12.6 | 10.9 | 0.254 | 15.6 | 12.3 | 0.214 |
|  | CBD - PBO (T) | -8.6 | 9.0 | 0.341 | -14.7 | 10.9 | 0.185 | 1.2 | 12.3 | 0.926 |
|  | (BL – T CBD) – (BL – T PBO) | 8.6 | 9.9 | 0.39 | 2.1 | 13.9 | 0.883 | 14.4 | 13.9 | 0.315 |
| Calm | BL-T (CBD) | 21.9 | 8.9 | 0.019 | 22.4 | 13.0 | 0.1 | 21.4 | 9.6 | 0.04 |
|  | BL-T (PBO) | 30.3 | 8.9 | 0.002 | 11.4 | 13.6 | 0.413 | 49.2 | 9.1 | <0.001 |
|  | CBD -PBO (BL) | 2.5 | 10.8 | 0.818 | 22.9 | 13.3 | 0.093 | -21.2 | 15.6 | 0.186 |
|  | CBD - PBO (T) | 10.8 | 10.8 | 0.318 | 11.9 | 13.3 | 0.376 | 6.6 | 15.6 | 0.674 |
|  | (BL – T CBD) – (BL – T PBO) | -8.4 | 12.6 | 0.512 | 11.0 | 18.8 | 0.564 | -27.8 | 13.2 | 0.051 |
| Comfortable | BL-T (CBD) | 28.1 | 8.6 | 0.002 | 39.8 | 11.8 | 0.003 | 13.8 | 11.8 | 0.257 |
|  | BL-T (PBO) | 34.0 | 8.6 | <0.001 | 21.7 | 12.4 | 0.095 | 46.3 | 11.2 | <0.001 |
|  | CBD -PBO (BL) | -1.4 | 9.9 | 0.885 | 21.2 | 13.0 | 0.111 | -28.6 | 12.7 | 0.031 |
|  | CBD - PBO (T) | 4.5 | 9.9 | 0.651 | 3.2 | 13.0 | 0.81 | 3.9 | 12.7 | 0.763 |
|  | (BL – T CBD) – (BL – T PBO) | -5.9 | 12.2 | 0.632 | 18.1 | 17.1 | 0.303 | -32.5 | 16.3 | 0.062 |
| Depressed | BL-T (CBD) | 2.7 | 4.5 | 0.55 | -4.0 | 4.4 | 0.373 | 10.9 | 8.3 | 0.209 |
|  | BL-T (PBO) | -4.6 | 4.5 | 0.312 | -6.5 | 4.6 | 0.175 | -2.7 | 7.9 | 0.737 |
|  | CBD -PBO (BL) | 4.6 | 4.5 | 0.31 | 2.1 | 4.5 | 0.645 | 7.9 | 8.1 | 0.336 |
|  | CBD - PBO (T) | -2.7 | 4.5 | 0.547 | -0.4 | 4.5 | 0.931 | -5.7 | 8.1 | 0.491 |
|  | (BL – T CBD) – (BL – T PBO) | 7.3 | 6.3 | 0.257 | 2.5 | 6.4 | 0.701 | 13.6 | 11.5 | 0.253 |
| Explorative | BL-T (CBD) | -37.9 | 6.7 | <0.001 | -54.1 | 9.3 | <0.001 | -18.1 | 7.2 | 0.022 |
|  | BL-T (PBO) | -21.6 | 6.7 | 0.003 | -30.6 | 9.8 | 0.005 | -12.5 | 6.8 | 0.085 |
|  | CBD -PBO (BL) | -4.6 | 6.7 | 0.497 | -7.5 | 9.6 | 0.439 | -1.2 | 7.0 | 0.863 |
|  | CBD - PBO (T) | 11.8 | 6.7 | 0.08 | 16 | 9.6 | 0.104 | 4.4 | 7.0 | 0.537 |
|  | (BL – T CBD) – (BL – T PBO) | -16.4 | 9.4 | 0.091 | -23.5 | 13.5 | 0.097 | -5.6 | 9.9 | 0.58 |
| Lethargic | BL-T (CBD) | 15.5 | 8.8 | 0.088 | 14.1 | 14.1 | 0.331 | 17.2 | 10.7 | 0.128 |
|  | BL-T (PBO) | 18.4 | 8.8 | 0.044 | 16.5 | 14.8 | 0.279 | 20.2 | 10.2 | 0.063 |
|  | CBD -PBO (BL) | 2.8 | 9.7 | 0.773 | 5.1 | 15.6 | 0.749 | -0.4 | 11.4 | 0.973 |
|  | CBD - PBO (T) | 5.7 | 9.7 | 0.558 | 7.5 | 15.6 | 0.636 | 2.7 | 11.4 | 0.815 |
|  | (BL – T CBD) – (BL – T PBO) | -2.9 | 12.5 | 0.818 | -2.4 | 20.4 | 0.908 | -3.1 | 14.8 | 0.837 |
| Nervous | BL-T (CBD) | -28 | 7.8 | <0.001 | -17.4 | 9.9 | 0.096 | -41 | 11.0 | 0.002 |
|  | BL-T (PBO) | -39.1 | 7.8 | <0.001 | -25.3 | 10.4 | 0.025 | -52.9 | 10.4 | <0.001 |
|  | CBD -PBO (BL) | 5.6 | 9.5 | 0.556 | -3.7 | 10.2 | 0.72 | 17.0 | 14.2 | 0.241 |
|  | CBD - PBO (T) | -5.5 | 9.5 | 0.568 | -11.6 | 10.2 | 0.262 | 5.1 | 14.2 | 0.722 |
|  | (BL – T CBD) – (BL – T PBO) | 11.1 | 11.0 | 0.318 | 7.9 | 14.4 | 0.589 | 11.9 | 15.1 | 0.442 |
| Reactive | BL-T (CBD) | -11.2 | 7.5 | 0.143 | -10.5 | 11.5 | 0.375 | -12.2 | 9.8 | 0.233 |
|  | BL-T (PBO) | -13.4 | 7.5 | 0.081 | -11.0 | 12.1 | 0.374 | -15.8 | 9.3 | 0.108 |
|  | CBD -PBO (BL) | -7.3 | 9.4 | 0.444 | -9.0 | 13.2 | 0.501 | -4.3 | 13.6 | 0.756 |
|  | CBD - PBO (T) | -9.5 | 9.4 | 0.319 | -9.5 | 13.2 | 0.475 | -8.0 | 13.6 | 0.564 |
|  | (BL – T CBD) – (BL – T PBO) | 2.2 | 10.6 | 0.837 | 0.5 | 16.7 | 0.974 | 3.7 | 13.6 | 0.789 |
| Relaxed | BL-T (CBD) | 23.6 | 7.7 | 0.004 | 35.2 | 11.2 | 0.005 | 9.5 | 9.8 | 0.347 |
|  | BL-T (PBO) | 33.6 | 7.7 | <0.001 | 26.3 | 11.7 | 0.037 | 41.0 | 9.3 | <0.001 |
|  | CBD -PBO (BL) | -6.3 | 9.4 | 0.507 | 15.4 | 12.8 | 0.237 | -32.4 | 11.9 | 0.01 |
|  | CBD - PBO (T) | 3.7 | 9.4 | 0.694 | 6.5 | 12.8 | 0.614 | -0.9 | 11.9 | 0.938 |
|  | (BL – T CBD) – (BL – T PBO) | -10.0 | 10.9 | 0.365 | 8.9 | 16.2 | 0.59 | -31.4 | 13.5 | 0.033 |
| Restless | BL-T (CBD) | -34.5 | 8.8 | <0.001 | -40.5 | 13.3 | 0.007 | -27.2 | 10.0 | 0.015 |
|  | BL-T (PBO) | -34.3 | 8.8 | <0.001 | -21.2 | 14.0 | 0.145 | -47.4 | 9.5 | <0.001 |
|  | CBD -PBO (BL) | 3.9 | 10.6 | 0.717 | -18.5 | 13.6 | 0.182 | 30.3 | 14.6 | 0.048 |
|  | CBD - PBO (T) | 4.1 | 10.6 | 0.701 | 0.7 | 13.6 | 0.957 | 10.2 | 14.6 | 0.491 |
|  | (BL – T CBD) – (BL – T PBO) | -0.2 | 12.4 | 0.985 | -19.3 | 19.3 | 0.331 | 20.1 | 13.8 | 0.164 |
| Sad | BL-T (CBD) | -2.1 | 6.7 | 0.756 | -11.8 | 7.0 | 0.108 | 9.7 | 11.7 | 0.418 |
|  | BL-T (PBO) | -21.4 | 6.7 | 0.003 | 21.7 | 7.3 | 0.008 | -21.0 | 11.1 | 0.076 |
|  | CBD -PBO (BL) | -1.1 | 6.7 | 0.877 | -7.6 | 7.9 | 0.339 | 6.8 | 11.4 | 0.555 |
|  | CBD - PBO (T) | -20.3 | 6.7 | 0.004 | -17.5 | 7.9 | 0.032 | -23.9 | 11.4 | 0.044 |
|  | (BL – T CBD) – (BL – T PBO) | 19.2 | 9.5 | 0.051 | 9.9 | 10.1 | 0.341 | 30.7 | 16.1 | 0.074 |
| Stressed | BL-T (CBD) | -38.4 | 7.6 | <0.001 | -23.2 | 9.8 | 0.029 | -57.0 | 9.5 | <0.001 |
|  | BL-T (PBO) | -56.3 | 7.6 | <0.001 | -42.0 | 10.3 | <0.001 | -70.6 | 9.0 | <0.001 |
|  | CBD -PBO (BL) | 3.5 | 9.5 | 0.715 | -8.3 | 10.5 | 0.437 | 17.6 | 12.4 | 0.166 |
|  | CBD - PBO (T) | -14.4 | 9.5 | 0.136 | -27.1 | 10.5 | 0.014 | 4.0 | 12.4 | 0.749 |
|  | (BL – T CBD) – (BL – T PBO) | 17.9 | 10.7 | 0.103 | 18.8 | 14.2 | 0.2 | 13.6 | 13.1 | 0.314 |
| Tense | BL-T (CBD) | -27.0 | 6.8 | <0.001 | -14.0 | 8.3 | 0.11 | -42.9 | 10.1 | <0.001 |
|  | BL-T (PBO) | -39.7 | 6.8 | <0.001 | -31.9 | 8.7 | 0.002 | -47.5 | 9.6 | <0.001 |
|  | CBD -PBO (BL) | 2.7 | 10.1 | 0.795 | -9.0 | 10.1 | 0.377 | 17.4 | 14.4 | 0.237 |
|  | CBD - PBO (T) | -10.1 | 10.1 | 0.325 | -27.0 | 10.1 | 0.011 | 12.8 | 14.4 | 0.382 |
|  | (BL – T CBD) – (BL – T PBO) | 12.7 | 9.7 | 0.196 | 17.9 | 12.1 | 0.154 | 4.6 | 13.9 | 0.745 |
| Uncomfortable | BL-T (CBD) | -40.3 | 7.0 | <0.001 | -30.5 | 9.6 | 0.005 | -52.3 | 8.9 | <0.001 |
|  | BL-T (PBO) | -55.7 | 7.0 | <0.001 | -41.7 | 10.1 | <0.001 | -69.8 | 8.4 | <0.001 |
|  | CBD -PBO (BL) | 2.5 | 10.0 | 0.805 | -14.9 | 11.4 | 0.201 | 23.2 | 12.8 | 0.08 |
|  | CBD - PBO (T) | -12.9 | 10.0 | 0.201 | -26.1 | 11.4 | 0.028 | 5.8 | 12.8 | 0.654 |
|  | (BL – T CBD) – (BL – T PBO) | 15.4 | 9.9 | 0.13 | 11.2 | 13.9 | 0.431 | 17.4 | 12.2 | 0.171 |
| PC1 | BL-T (CBD) | -2.4 | 0.5 | <0.001 | -2.3 | 0.8 | 0.013 | -2.6 | 0.7 | <0.001 |
|  | BL-T (PBO) | -3.2 | 0.5 | <0.001 | -2.4 | 0.9 | 0.014 | -4.1 | 0.6 | <0.001 |
|  | CBD -PBO (BL) | 0.1 | 0.7 | 0.919 | -1.1 | 0.9 | 0.23 | 1.5 | 1.0 | 0.1 |
|  | CBD - PBO (T) | -0.1 | 0.7 | 0.333 | -1.2 | 0.9 | 0.192 | 0.1 | 1.0 | 0.943 |
|  | (BL – T CBD) – (BL – T PBO) | 0.8 | 0.8 | 0.308 | 0.1 | 1.2 | 0.934 | 1.5 | 0.9 | 0.128 |
